# Phytochemical Profiling and Antioxidant Activity of *Justicia thunbergioides* (Lindau) Leonard (Acanthaceae): A Promising Source of Therapeutic Metabolites

**DOI:** 10.3390/ph19030486

**Published:** 2026-03-16

**Authors:** Laryssa Rosset Provensi, Marcos Rodrigo Beltrão Carneiro, Alisson Martins-Oliveira, André Luiz Meleiro Porto, Eric de Souza Gil, Josana de Castro Peixoto, Lucimar Pinheiro Rosseto

**Affiliations:** 1 Biodiversity Research Laboratory, Evangelical University of Goias, Anápolis 75083-515, GO, Brazil; laryssarosset@gmail.com (L.R.P.); marcos.rodrigo@ueg.br (M.R.B.C.); alissonmartini@yahoo.com.br (A.M.-O.); josana.peixoto@unievangelica.edu.br (J.d.C.P.); 2 Campus Central, State University of Goiás, Anápolis 75132-400, GO, Brazil; 3 Institute of Chemistry, University of São Paulo, São Carlos Campus, São Carlos 13566-590, SP, Brazil; almporto@iqsc.usp.br; 4 Faculty of Pharmacy, Federal University of Goiás (UFG), Goiânia 74690-900, GO, Brazil; ericsgil@ufg.br

**Keywords:** phytochemical profiling, *Justicia*, antioxidant activity

## Abstract

**Background/Objectives**: Medicinal plants are widely investigated due to their rich content of biologically active secondary metabolites with potential therapeutic applications. This study aimed to investigate the phytochemical profile and antioxidant activity of extracts with different polarities obtained from *Justicia thunbergioides* (Lindau) Leonard (Acanthaceae). **Methods**: Phytochemical screening was initially performed through qualitative analysis, followed by fractionation and characterization of dichloromethane and methanolic extracts using thin-layer chromatography (TLC) and gas chromatography–mass spectrometry (GC–MS). Antioxidant activity was evaluated using the 2,2-diphenyl-1-picrylhydrazyl (DPPH) radical scavenging assay and electrochemical techniques. **Results**: GC–MS analysis of the dichloromethane extract revealed a chemically diverse composition, including compounds such as spathulenol, vitamin E, sesamin, squalene, and β-sitosterol, which are widely reported in the literature for their antioxidant and bioactive properties. The methanolic extract exhibited a distinct chemical profile, with a predominance of phenolic and redox-active compounds. DPPH assays demonstrated that the methanolic extract showed the highest radical scavenging capacity in a concentration-dependent manner, whereas the dichloromethane and hexane extracts required higher concentrations to achieve moderate antioxidant effects. Electrochemical analyses indicated that the methanolic extract is rich in electroactive metabolites capable of partially reversible electron transfer, consistent with its enhanced antioxidant performance. **Conclusions**: Collectively, these findings highlight the antioxidant efficacy of the polar extracts from *J. thunbergioides* and contribute to a better understanding of the bioactivity of their phytochemical constituents.

## 1. Introduction

Compared with synthetic drugs, medicinal plants play a crucial role in healthcare systems, particularly because of their affordability and generally lower incidence of side effects. A significant number of modern pharmaceuticals originate from natural sources, many of which have served as therapeutic agents for a long time [1]. The World Health Organization (WHO) estimates that approximately 80% of the global population relies on traditional medicines derived from medicinal plants, especially in underserved regions [2]. In line with this perspective, more than 350,000 medicinal plant species have been cataloged, highlighting their relevance as sources for the development of novel bioactive and therapeutic agents [3].

In this context, the Brazilian Cerrado represents the second largest biome in Brazil and is characterized by a remarkable diversity of native plant species, many of which exhibit considerable medicinal potential, largely attributed to their production of bioactive secondary metabolites [4,5] such as alkaloids [6], coumarins [7], flavonoids [8], tannins [9], terpenoids [10] and saponins [11]. This extensive chemical diversity positions the Cerrado as a strategic reservoir for the discovery of pharmacologically relevant natural compounds.

A growing body of evidence demonstrates that several plant species native to the Cerrado can modulate physiological and biochemical pathways, resulting in significant therapeutic effects related to oxidative stress, inflammatory processes, and microbial infections [11,12,13,14,15]. Therefore, the phytochemical richness of the Cerrado extends beyond biodiversity itself and represents a valuable source of bioactive molecules with potential pharmaceutical applications, underscoring the importance of continued phytochemical and pharmacological investigations.

In agreement with these observations, oxidative stress represents a critical imbalance between elevated reactive oxygen species (ROS) production and impaired endogenous antioxidant activity systems, contributing to the onset and progression of several chronic illnesses [16]. In fact, most human diseases, including cancer and cardiovascular disorders, are associated with the overproduction of free radicals [17]. Previous studies have consistently shown the antioxidant and antibacterial properties of flavonoid-rich plants and their role in mitigating chronic diseases [18,19]. Within this context, members of the Acanthaceae family, particularly species of the *Justicia* genus, have shown a broad spectrum of phytoconstituents with promising pharmacological profiles [20].

Owing to the chemical diversity and multifaceted biological activity of *Justicia* species, a prior study with *Justicia adhatoda* extract revealed potential anti-inflammatory, analgesic, antibacterial and antioxidant activities [20]. Indeed, *Justicia secunda* methanolic leaf extracts have been shown to exert significant anti-inflammatory, hepatoprotective, nephroprotective, and immunomodulatory effects in vitro and in vivo models [21]. Emerging studies also highlight the gastroprotective properties of *Justicia pectoralis*, as evidenced by notable protective effects against chemically induced gastric lesions [22]. Despite these advances, limited information is available regarding the phytochemical composition and antioxidant potential of *Justicia thunbergioides*. Therefore, the present study aimed to investigate the phytochemical profile and the antioxidant activity of *J. thunbergioides* (Lindau) Leonard (Acanthaceae) leaves.

## 2. Results

### 2.1. Phytochemical Analysis

Preliminary phytochemical screening of *J. thunbergioides* leaf extract was conducted to identify the presence of various bioactive compounds, including anthraquinones, alkaloids, coumarins, tannins, saponins, flavonoids, and terpenoids. The results revealed a strong presence of alkaloids, as confirmed by six distinct qualitative assays. Positive results were obtained in terms of terpenoids through three different tests. Flavonoids were consistently detected across multiple assays. Although tannins, coumarins, and saponins have been identified, no evidence of anthraquinone derivatives was observed in the tested samples. Flavonoids, alkaloids, terpenoids and tannins were the most abundant classes of compounds found in the leaves of *J. thunbergioides* (Table 1).

### 2.2. Compositional Analysis of the Dichloromethane Extracts of J. thunbergioides by GC-MS

**Figure 1:**
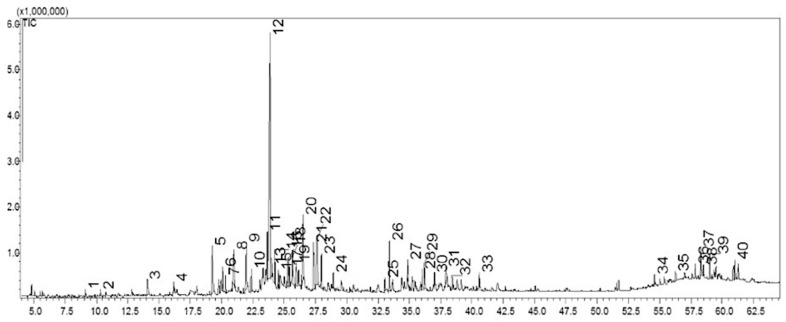
Chromatogram of the ethyl acetate fraction obtained from the dichloromethane extract of *J. thunbergioides*. Chromatographic peaks are labeled according to increasing retention time. Analyses were carried out on a DB-5MS capillary column. The oven temperature was programmed from 60 to 250 °C at a rate of 5 °C min^−1^. The injector temperature was set at 200 °C. The carrier gas flow rate was 1 mL min^−1^, with a split ratio of 20:1. The total analysis time was 60 min.

To our knowledge, studies addressing the chemical composition of crude extracts from *J. thunbergioides* have not been reported in the literature. Gas chromatography–mass spectrometry (GC–MS) analysis allowed the detection of 40 chemical constituents in the ethyl acetate fraction (JTFDA) derived from the dichloromethane extract. Among the 40 chromatographic peaks detected, 16 secondary metabolites were unambiguously identified (Figure 1). The retention times (RTs) for each compound are summarized in Table 2. In the present study, the major constituent (33.80%, peak 12) could not be identified, as its mass spectrum did not match any entries in the consulted libraries.

The identified compounds in the ethyl acetate fraction obtained from the dichloromethane extract belong to the following chemical classes: terpenes (1,8-cineole, *p*-cymen-8-ol,2,6-dimethylocta-1,7-diene-3,6-diol, *trans*-*p*-menth-6-en-2,8-diol, 8-Hydroxycarvotanacetone, spathulenol, oplopanone, hydrocarbons (decane); ketones (cyclooctanone); fatty alcohols (1-hexacosanol and octacosanol); tocopherols (α-tocopherol); lignans (sesamin); and steroids (β-sitosterol), (Figure 1).

**Figure 2:**
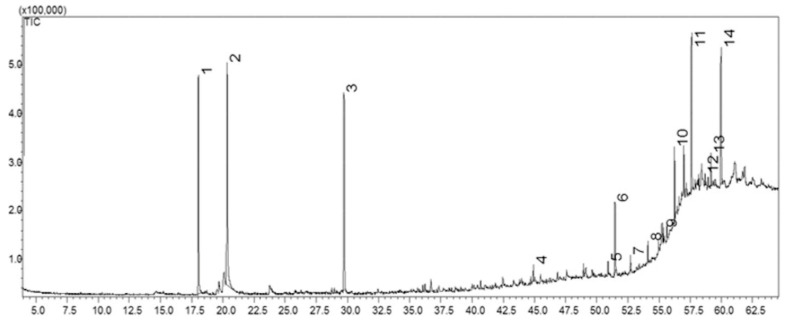
Chromatographic profile of the dichloromethane-soluble fraction obtained from the dichloromethane extract of *J. thunbergioides*. Individual components are indicated according to increasing retention times. Analyses were carried out on a DB-5MS capillary column. The oven temperature was programmed from 60 to 250 °C at a rate of 5 °C min^−1^. The injector temperature was set at 200 °C. The carrier gas flow rate was 1 mL min^−1^, with a split ratio of 20:1. The total analysis time was 60 min.

On the other hand, GC–MS analysis revealed the presence of 14 chemical constituents in the dichloromethane partition (JTFDD) obtained from the dichloromethane extract of *J. thunbergioides*. Among the 14 chromatographic peaks detected, 13 secondary metabolites were identified (Figure 2). The retention times (RTs) are presented in Table 3. On the basis of the results of the GC–MS peak area normalization, the most abundant constituents identified were the monoterpene endoperoxides ascaridole (19.81%) and isoascaridole (26.38%), as well as the sesquiterpene β-oplopenone (17.26%), corresponding to peaks 1, 2, and 3, respectively. Additional compounds were assigned to the terpene class (squalene), hydrocarbons (pentacosane, hexacosane, heptacosane, and nonacosane), and aldehydes (octacosanal, nonacosanal, and triacontanal). The mass spectra obtained from the GC-MS analyses are available in the Supplementary Materials (Figures S1 and S2).

### 2.3. Characterization of Secondary Metabolites of the Methanolic Extract of J. thunbergioides

**Figure 3:**
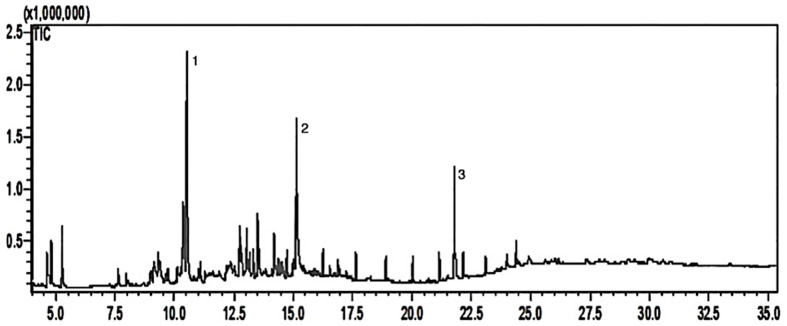
Chromatogram of the ethyl acetate fraction obtained from the methanolic extract of *J. thunbergioides*. Individual components are indicated according to increasing retention times. Analyses were performed using a DB-5MS capillary column (crosslinked 5% phenyl-methylpolysiloxane). The oven temperature was programmed from 60 to 250 °C at a rate of 2 °C min^−1^. The injector temperature was set at 200 °C. The carrier gas flow rate was 1 mL min^−1^, with a split ratio of 20:1. The total analysis time was 35 min and the inlet pressure was maintained at 24.0 kPa.

To obtain secondary metabolites from the methanolic extract of *J. thunbergioides*, an acid–base fractionation procedure was applied, with the aim of separating alkaloids and coumarins. The resulting chloroform (F1), ethyl acetate (F2), and aqueous (F3) fractions were subsequently evaluated via thin-layer chromatography (TLC) and gas chromatography–mass spectrometry (GC–MS). The ethyl acetate fraction (F2) was selected for detailed analysis because of its positive response to the visualization reagents employed (UV irradiation at 254 and 366 nm and anisaldehyde spray reagent), as well as its greater yield (2 g) than the chloroform fraction (F1). The GC–MS total ion chromatogram of the ethyl acetate fraction (F2) derived from the methanolic extract of *J. thunbergioides* is presented in Figure 3. The chromatographic profile revealed three prominent peaks of interest at retention times t_R1_ = 10.56 min, t_R2_ = 15.18 min, and t_R3_ = 21.80 min. The signal observed at t_R3_ = 21.80 min was disregarded, as spectral library matching indicated that it corresponded to a phthalate contaminant, most likely introduced during solvent handling.

Analysis of the chromatograms obtained from fractions F57–F63, derived from the ethyl acetate partition (F2) of the methanolic extract of *J. thunbergioides*, indicated the partial isolation of a single compound, with minor contamination. The compound was identified through comparison of its mass spectrum with data reported in the literature and was assigned as an isoascaridole [23]. It was also detected in the dichloromethane extract, accounting for 26.38% of the total composition.

On the basis of TLC monitoring, fractions F201–F210 derived from the ethyl acetate partition (F2) were combined and further examined via GC–MS. The corresponding total ion chromatogram revealed the presence of a single compound eluting at t_R_ = 11.74 min. Comparison of its mass spectrum with published data indicated a 96% match with butylated hydroxytoluene (BHT) [24]. The presence of BHT is attributed to its use as a stabilizer in ethyl acetate and is therefore considered a chromatographic contaminant.

### 2.4. Antioxidant Activity

The 2,2-diphenyl-1-picrylhydrazyl (DPPH) radical scavenging assay is a widely recognized and reliable method for evaluating the antioxidant activity of plant extracts. Its popularity stems from its simplicity, rapid execution, and reproducibility. The DPPH radical itself is considered an effective indicator of antioxidant capacity because of its strong ability to accept hydrogen atoms from antioxidant compounds. In our study, we evaluated the DPPH radical scavenging activity of both polar and apolar extracts derived from the leaves of *J. thunbergioides*.

**Figure 4:**
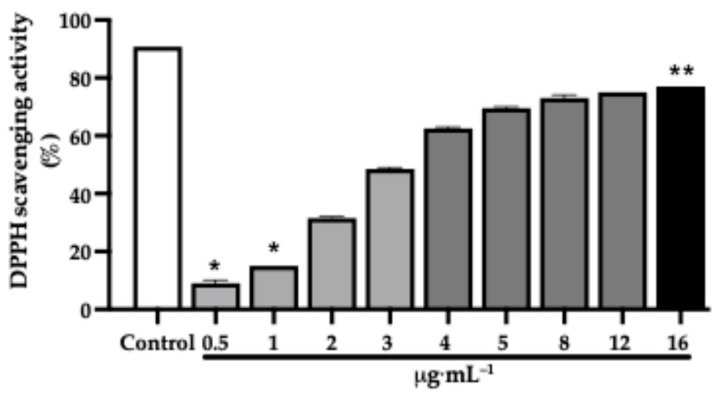
Concentration-dependent 2,2-diphenyl-1-picrylhydrazyl (DPPH) radical scavenging activities of the *J. thunbergioides* methanolic extract. The bar graphs with asterisks (*) denote a significant difference compared with the positive control (gallic acid, 10 µmol) (*p*  ≤  0.05), and those with asterisks (**) denote a significant difference compared with 0,5 and 1 μg·mL^−1^ (*p*  ≤  0.05). The Kruskal–Wallis test with Dunnett’s multiple comparison test was used. The data are presented as the means ± SDs of 2 independent experiments performed in duplicate.

The methanolic extract exhibited a concentration-dependent DPPH radical scavenging activity (Figure 4). At lower concentrations (0.5 and 1 µg·mL^−1^), the extract showed minimal scavenging activity, which was significantly lower than that of the control. From 2 µg·mL^−1^ onward, a progressive increase in antioxidant activity was observed, with scavenging values exceeding 60% at concentrations ≥ 4 µg·mL^−1^. The highest scavenging activity was recorded at 16 µg·mL^−1^, which was significantly greater than that observed at the lowest tested concentrations. The IC_50_ value of the methanolic extract was 3.2 µg·mL^−1^, indicating strong radical scavenging capacity. Under the same experimental conditions, the positive control, gallic acid, presented an IC_50_ value of 4.2 µg·mL^−1^, allowing direct comparison of antioxidant potency.

**Figure 5:**
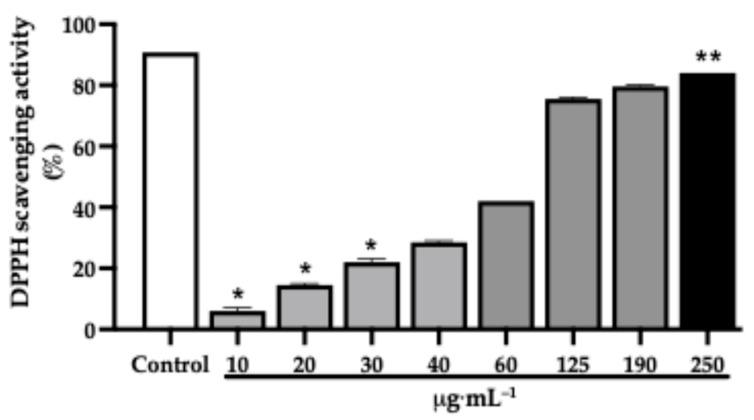
Concentration-dependent 2,2-diphenyl-1-picrylhydrazyl (DPPH) radical scavenging activities of the *J. thunbergioides* dichloromethanic extract. The bar graphs with asterisks (*) denote a significant difference compared with the positive control (gallic acid, 10 µmol) (*p*  ≤  0.05), and the bar graphs with asterisks (**) denote a significant difference compared with the 10 and 20 μg·mL^−1^ groups (*p*  ≤  0.05). The Kruskal–Wallis test with Dunnett’s multiple comparison test was used. The data are presented as the means ± SDs of 2 independent experiments performed in duplicate.

The dichloromethane extract also exhibited a concentration-dependent increase in DPPH radical scavenging activity (Figure 5). At lower concentrations (10–20 µg·mL^−1^), scavenging activity remained below 15%, increasing to approximately 40% at 60 µg·mL^−1^. More pronounced radical scavenging was observed at higher concentrations, exceeding 70% at 125–190 µg·mL^−1^ and reaching a maximum of over 80% at 250 µg·mL^−1^. The IC_50_ value was determined to be 78.5 µg·mL^−1^, with an antioxidant activity index (AAI) of 0.25 (Table 4).

**Figure 6:**
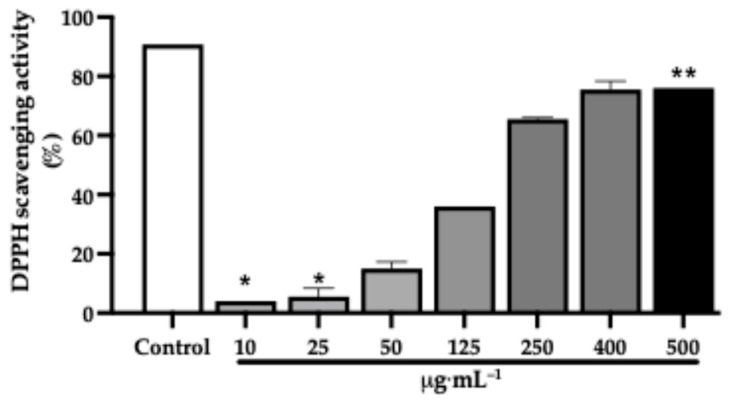
Concentration-dependent 2,2-diphenyl-1-picrylhydrazyl (DPPH) radical scavenging activities of the *J. thunbergioides* hexanic extract. The bar graphs with asterisks (*) denote a significant difference compared with the positive control (gallic acid, 10 µmol) (*p*  ≤  0.05), and those with asterisks (**) denote a significant difference compared with the 10 and 25 μg·mL^−1^ treatments (*p*  ≤  0.05). The Kruskal–Wallis test with Dunnett’s multiple comparison test was used. The data are presented as the means ± SDs of 2 independent experiments performed in duplicate.

In addition, the hexane extract, at lower concentrations (10 and 25 µg·mL^−1^), exhibited minimal activity, with statistically significant differences compared with the control (*p* < 0.05), (Figure 6). From 50 to 125 µg·mL^−1^, a gradual increase in DPPH inhibition was observed, reaching approximately 35% at 125 µg·mL^−1^. At 250 µg·mL^−1^, scavenging activity exceeded 65%, while the highest inhibition was recorded at 500 µg·mL^−1^, where values surpassed 75% and were significantly different from all other tested concentrations (*p* < 0.05). The IC_50_ value was calculated as 186.3 µg·mL^−1^, with an antioxidant activity index (AAI) of 0.10 (Table 4).

Overall, the lower antioxidant activity of the hexane extract than that of the methanolic extract is likely related to the distinct polarity of the extraction solvents. Since phenolic compounds are predominantly polar and represent the major contributors to free radical scavenging capacity, they are more efficiently extracted in methanol. Consequently, the methanolic extract displayed superior DPPH radical scavenging activity, which was consistent with its higher phenolic content and enhanced antioxidant potential.

**Figure 7:**
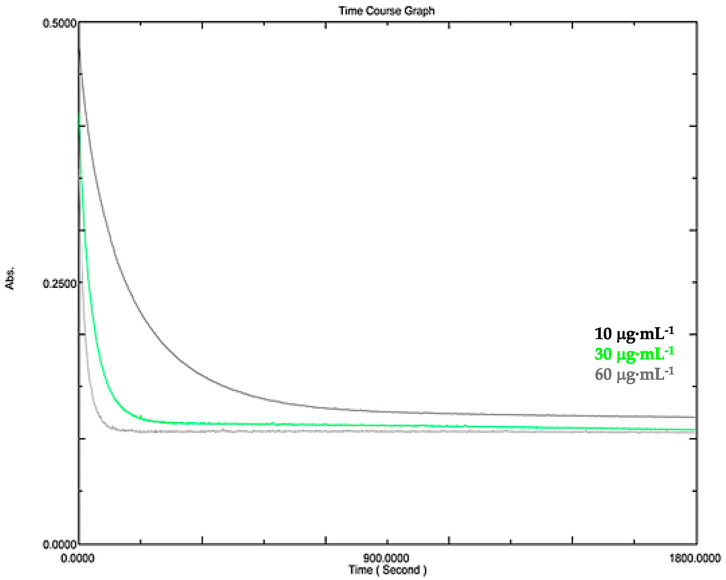
Kinetics of the decrease in 2,2-diphenyl-1-picrylhydrazyl (DPPH) absorbance at 517 nm in the presence of *J. thunbergioides* leaf methanolic extract at 10, 30 and 60 µg·mL^−1^.

**Figure 8:**
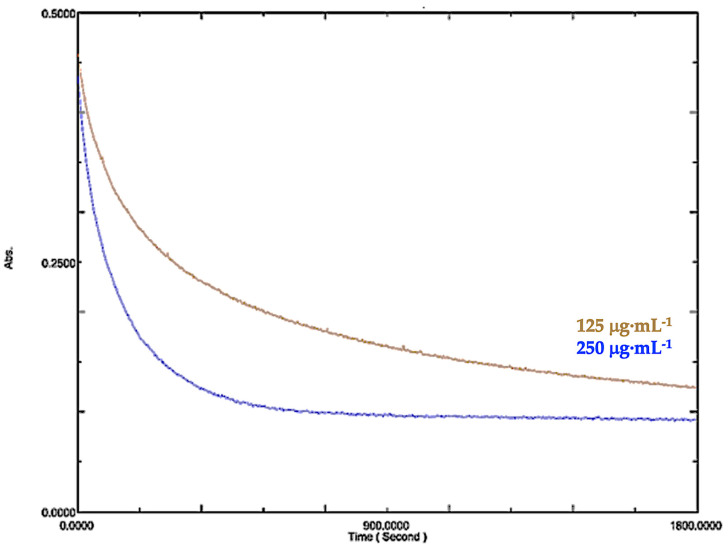
Kinetics of the decrease in 2.2-diphenyl-1-picrylhydrazyl (DPPH) absorbance at 517 nm in the presence of *J. thunbergioides* leaf dichloromethane extract at 125 and 250 µg·mL^−1^.

The DPPH kinetic assay demonstrated a concentration-dependent reduction in the absorbance of the methanolic extract (Figure 7). The 60 µg·mL^−1^ dose resulted in the most rapid decrease, indicating strong and immediate antioxidant activity. At 30 µg·mL^−1^, the decrease was moderate and slightly delayed, whereas the 10 µg·mL^−1^ dose exhibited a gradual and less pronounced effect. These findings suggest that higher extract concentrations result in faster and more efficient free radical scavenging. The dichloromethane extract exhibited a time- and concentration-dependent reduction in DPPH absorbance (Figure 8). At 250 µg·mL^−1^, the extract showed faster and more intense antioxidant activity than at 125 µg·mL^−1^, indicating greater radical scavenging efficiency at higher concentrations.

**Figure 9:**
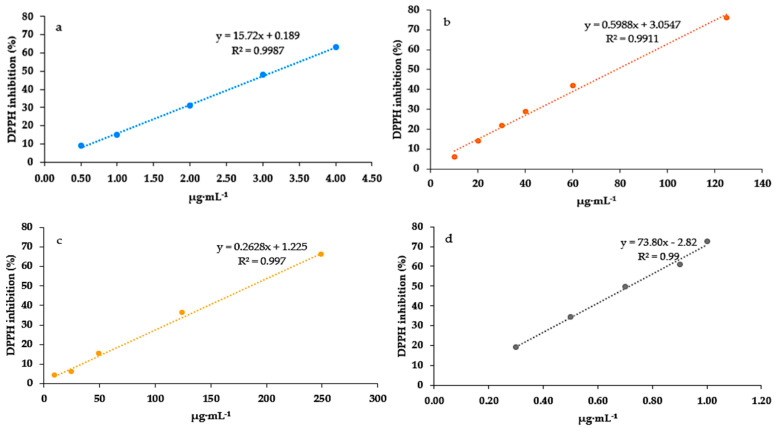
Linear correlation between 2,2-diphenyl-1-picrylhydrazyl (DPPH) radical scavenging activity and the concentration-dependent response of *J. thunbergioides* extracts with different polarities. (**a**) methanolic extract, (**b**) dichloromethanic extract, (**c**) hexanic extract and (**d**) gallic acid (positive control).

DPPH inhibition assays of the three extracts of *J. thunbergioides* revealed distinct antioxidant capacities. The methanolic extract (Figure 9a) exhibited the highest activity, with a strong linear correlation (R^2^ = 0.9987), indicating a potent radical scavenging efficiency. The dichloromethane extract (Figure 9b) showed moderate activity (R^2^ = 0.9911), whereas the hexane extract (Figure 9c) demonstrated the weakest antioxidant response, despite the evident linearity (R^2^ = 0.997). The correlation coefficient (R^2^) for gallic acid used with the positive control exceeded 0.98 (Figure 9d). The linear calibration curves demonstrated excellent accuracy and precision, underscoring the robustness of the analytical method, and all extracts yielded correlation coefficients (R^2^) greater than 0.98, confirming the method’s strong linearity and reproducibility across the concentration range (Figure 9a–d). These results suggested that antioxidant activity was strongly associated with extract polarity, with the more polar extract having the greatest effect.

### 2.5. Voltammetric Analysis

**Figure 10:**
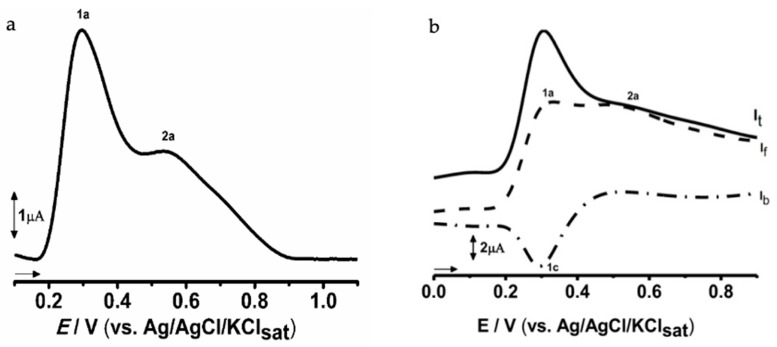
Differential pulse voltammetry (DPV) and square wave voltammetry (SWV) profiles of the methanolic extract of *J. thunbergioides*. The voltammograms were obtained from 10 µg·mL^−1^ solutions. (**a**) DPV voltammogram showing two main anodic peaks (1a and 2a), attributed to the oxidation of electroactive antioxidant constituents in the extract. (**b**) SWV voltammogram displaying the total current (It), forward current (If), and backward current (Ib) components, with corresponding anodic (1a, 2a) and cathodic (1c) peaks, indicating the redox behavior of phenolic compounds.

To evaluate the redox profile of *J. thunbergioides*, electrochemical experiments were performed with 10 µg·mL^−1^ methanolic extract. Differential pulse voltammetry of the methanolic extract of *J. thunbergioides* revealed the occurrence of two successive anodic oxidation events (Figure 10). The first oxidation peak, observed at approximately Ep_1a_ = +0.25 V vs. Ag/AgCl, exhibited the highest current intensity, suggesting the presence of electroactive compounds with strong reducing capacity, likely phenolic or flavonoid constituents with well-recognized antioxidant potential. A second anodic peak, detected at approximately Ep_2a_ = +0.45 V, displayed a lower current response, which may correspond to the oxidation of less reactive compounds or secondary oxidation of intermediates formed during the first process. The electrochemical profile therefore highlighted the presence of redox-active molecules in the extract, which is consistent with the antioxidant activity previously reported in this study.

In this regard, the square wave voltammogram of the methanolic extract of *J. thunbergioides* (Figure 10) revealed two main anodic processes together with a cathodic return signal. In the total current (It) curve, a first anodic peak (1a) was observed at Ep_1a_ ≈ +0.3 V, followed by a second anodic peak (2a) at Ep_2a_ ≈ +0.50 V, which was less intense. The forward current (If) confirmed the characteristic increase associated with the oxidation of phenolic constituents, indicating that some of the present compounds undergo oxidation at relatively low potentials. On the other hand, the backward current (Ib) exhibited a cathodic peak (1c) at Ep_1c_ ≈ +0.30 V, which was consistent with the reduction in the electrogenerated products that probably formed during the first oxidation stage.

## 3. Discussion

While previous studies have documented the antioxidant activity of extracts from the *Justicia* leaf genus, this study is the first to report substantial concentration-dependent antioxidant effects in *J. thunbergioides* leaf extracts. Taking into account the extracts derived using solvents of varying polarity, the methanolic extract from *J. thunbergioides* presented the highest antioxidant activity (AAI > 2) and lower IC_50_ than did the dichloromethane and hexanic extracts. With respect to the Blois classification, the methanol extract had very strong antioxidant activity (IC_50_= 3.2 µg·mL^−1^; AAI = 6.15), similar to that of gallic acid (IC_50_= 4.2 µg·mL^−1^; AAI = 4.69). Compared with the other extracts, the dichloromethane fraction had a moderate antioxidant capacity (IC_50_= 78.5 µg·mL^−1^; AAI = 0.25), whereas the hexanic extract had the weakest antioxidant effect (IC_50_= 186.3 µg·mL^−1^; AAI = 0.10).

The potential antioxidant activity of the methanolic extract was likely correlated with the relatively high flavonoid content of this extract, as revealed by the preliminary phytochemical screening. In fact, earlier studies have established a positive correlation between antioxidant activity measured by the DPPH assay and total phenolic compound levels, especially those of flavonoids, which are affected by redox-based free radical neutralization [25,26]. This observation is well supported by extensive phytochemical literature, which has shown that polar solvents such as methanol are highly effective in extracting hydroxyl-rich phenolic compounds and flavonoids [27]. Flavonoids exert their antioxidant effects primarily through multiple mechanisms, including scavenging ROS and free radicals, chelating redox-active transition metals (such as Fe^2+^ and Cu^2+^), inhibiting lipid peroxidation, regulating mitochondrial dynamics, and reducing oxidative damage by modulating fusion and fission processes in cellular organelles [28,29].

In line with these findings, chromatographic analysis of the methanolic extract revealed the presence of the isoascaridole. Isoascaridole has been identified in plant extracts and essential oils exhibiting pronounced antioxidant activity in established in vitro assays, including DPPH-based methods [30,31]. Although the antioxidant effects have been primarily attributed to the complex phytochemical matrix rather than to isolated constituents, the repeated identification of isoascaridole in bioactive samples suggests its involvement in the observed effects.

The results obtained in this study are consistent with previous reports describing higher free radical scavenging capacity and reduced IC_50_ values in aqueous extracts of *Justicia adhatoda* [32]. Similar trends have been observed for other species within the genus; for instance, increased antioxidant activity in *Justicia spicigera* leaf extracts has been directly associated with elevated levels of phenolic compounds [33], while markedly enhanced free-radical scavenging capacities have also been reported for leaf extracts of *Justicia secunda* [34]. In addition, significant antioxidant effects have been described for the ethanolic extract of *Justicia pectoralis* Jacq. [35]. Collectively, these findings are in agreement with previous reports demonstrating that leaves from different Justicia species contain substantially higher levels of phenolic compounds and flavonoids compared with other plant parts, indicating that leaves represent the primary reservoirs of these bioactive metabolites and the most promising targets for antioxidant bioprospecting [36]. The absence of individual phenolic identification by GC–MS in the present study does not preclude their presence in the methanolic extract and instead reflects known analytical limitations of this technique for highly polar secondary metabolites. Taken together, the consistent antioxidant performance observed across *J. thunbergioides* and other *Justicia* species supports the hypothesis that shared biochemical features within the genus, particularly enrichment in phenolic and flavonoid compounds, underlie their antioxidant potential.

In contrast to the pronounced antioxidant activity of the methanolic extract, we detected moderate and weak concentration-dependent DPPH radical scavenging from the dichloromethane (moderately polar) and hexane (nonpolar) fractions, respectively. In fact, a previous phytochemical study confirmed the influence of solvent polarity on antioxidant efficacy; extracts obtained with dichloromethane and hexane presented total phenolic contents that were significantly lower than those of methanol extracts, and their DPPH IC_50_ values were notably higher [37]. These moderately polar and nonpolar solvents preferentially extract lipophilic constituents, including steroids, terpenoids and fatty acids, which are characterized by weaker radical neutralizing capacity, which may explain, at least in part, their markedly lower concentration-dependent DPPH antioxidant activity than the methanolic extract.

The qualitative and quantitative composition of plant extracts is known to vary according to species, harvesting conditions, extraction procedures, and the plant parts analyzed. In the present study, a diverse set of secondary metabolites with recognized antioxidant properties was identified in the ethyl acetate fraction (JTFDA) derived from the dichloromethane extract. Among these compounds, spathulenol is noteworthy due to its broad range of reported biological activities, including antinociceptive [38], antimicrobial [39], antiproliferative and antioxidant effects [40]. This sesquiterpene has also been previously identified in the essential oil obtained from the leaves of *Justicia schimperiana* [41], supporting its occurrence within the genus.

In addition, vitamin E (α-tocopherol), a well-established lipid-soluble antioxidant [42], has been reported in several species of the Acanthaceae family [43]. Beyond its antioxidant function, α-tocopherol has been associated with cancer-preventive properties [44] and exhibits a broad spectrum of biological activities, including antispasmodic, vasodilatory, antidiabetic, and hepatoprotective effects [45]. Sesamin, a furofuran-type lignan widely recognized as a major antioxidant constituent of *Sesamum indicum* L. seed oil [45], has also demonstrated protective effects against fluoride-induced liver damage in zebrafish through the attenuation of reactive oxygen species production and lipid peroxidation [46]. Notably, sesamin has been reported in other Justicia species, such as *J. hyssopifolia* and J. simplex, reinforcing its relevance as a bioactive lignan within the Acanthaceae family [47,48].

In addition to its widespread occurrence across several plant genera, including *Justicia*, β-sitosterol has been the subject of extensive investigation because of its broad spectrum of biological activities. In particular, this phytosterol is well recognized for its cholesterol-lowering capacity, as well as for its antioxidant and anti-inflammatory properties [49]. Accumulating evidence indicates that β-sitosterol may be beneficial in the management of hypercholesterolemia and hormone-related cancers [50,51,52].

Compounds such as spathulenol, sesamin, vitamin E (α-tocopherol), and β-sitosterol are known to contribute to antioxidant effects through complementary mechanisms, including attenuation of oxidative stress, inhibition of lipid peroxidation, and modulation of redox homeostasis. However, these metabolites are generally associated with moderate activity in single-electron transfer-based assays, such as DPPH, particularly when present in complex mixtures and at relatively low concentrations. Thus, the DPPH results are consistent with the phytochemical profile of the dichloromethane extract and support the notion that its antioxidant potential arises from the cumulative and possibly synergistic effects of multiple moderately active constituents rather than from a single highly potent antioxidant.

Consistent with this body of evidence, hexane extracts generally exhibit the lowest phenolic and flavonoid contents and, consequently, weaker antioxidant performance when compared with more polar extracts [53]. Furthermore, a solvent polarity-dependent pattern in the antioxidant activity of plant extracts has been widely reported, in which moderately polar and nonpolar solvents, such as dichloromethane and hexane, respectively, tend to yield lower antioxidant effects, whereas polar solvents are associated with substantially higher activity [54]. Collectively, these findings support the notion that nonpolar extracts display reduced radical scavenging capacity, primarily due to their limited ability to solubilize and extract phenolic and flavonoid compounds, which are recognized as key contributors to in vitro antioxidant activity.

The kinetic DPPH assay revealed a clear concentration-dependent and solvent-dependent response, wherein the increased extract dosage probably resulted in a higher content of antioxidant constituents, thereby facilitating more rapid and effective neutralization of DPHH free radicals. A previous study employing kinetic DPPH analysis revealed a stronger positive correlation between the antioxidant activity of the methanolic extract and its total phenolic content [55]. Taken together, these findings indicate that the methanolic extract of *J. thunbergioides* exhibits pronounced antioxidant activity in vitro. Although these results point to a potential role of this extract in mitigating oxidative processes, its ability to modulate endogenous antioxidant defense systems and protect against lipid peroxidation requires further investigation using appropriate cellular and in vivo models.

To further support the findings of this study, electrochemical assays were performed to characterize the redox profile of the *J. thunbergioides* methanolic extract. In the differential pulse voltammetry (DPV) analysis, variations in peak current intensities indicated that compounds oxidized at lower potentials were present at higher concentrations and/or exhibited greater electrochemical activity than those oxidized at higher potentials. This electrochemical response is consistent with the presence of redox-active secondary metabolites in the extract, corroborating its antioxidant potential.

The simultaneous detection of anodic peaks in It and If, together with a cathodic peak in Ib, indicates that the electroactive constituents undergo partially reversible redox processes. Such electrochemical behavior is characteristic of phenolic-rich mixtures, whose antioxidant properties are closely associated with their ability to donate electrons and participate in subsequent redox regeneration cycles.

Therefore, based on the current evidence, the enhanced antioxidant activity of the methanolic extract is most reasonably interpreted as resulting from a higher concentration of phenolic compounds rather than the presence of a single highly potent metabolite. Nonetheless, the contribution of specific redox-active constituents cannot be completely excluded and would require further targeted isolation and structural characterization.

## 4. Materials and Methods

### 4.1. Plant Collection

Mature leaves of *J. thunbergioides* were collected along the Corumba highway (15°56′05″ S, 48°50′46″ W) on the route toward Cocalzinho city (15°66′44″ S, 48°68′77″ W), Goiás, Brazil. The plants were deposited at the State University of Goiás Herbarium under numbers 723 and 16,819, where voucher specimens were obtained for identification and authentication. The fresh leaves showed no signs of necrosis, chlorosis, or fungal contamination. To ensure greater sample uniformity, collections were consistently carried out at approximately 10:00 a.m.

### 4.2. Extraction

**Figure 11:**
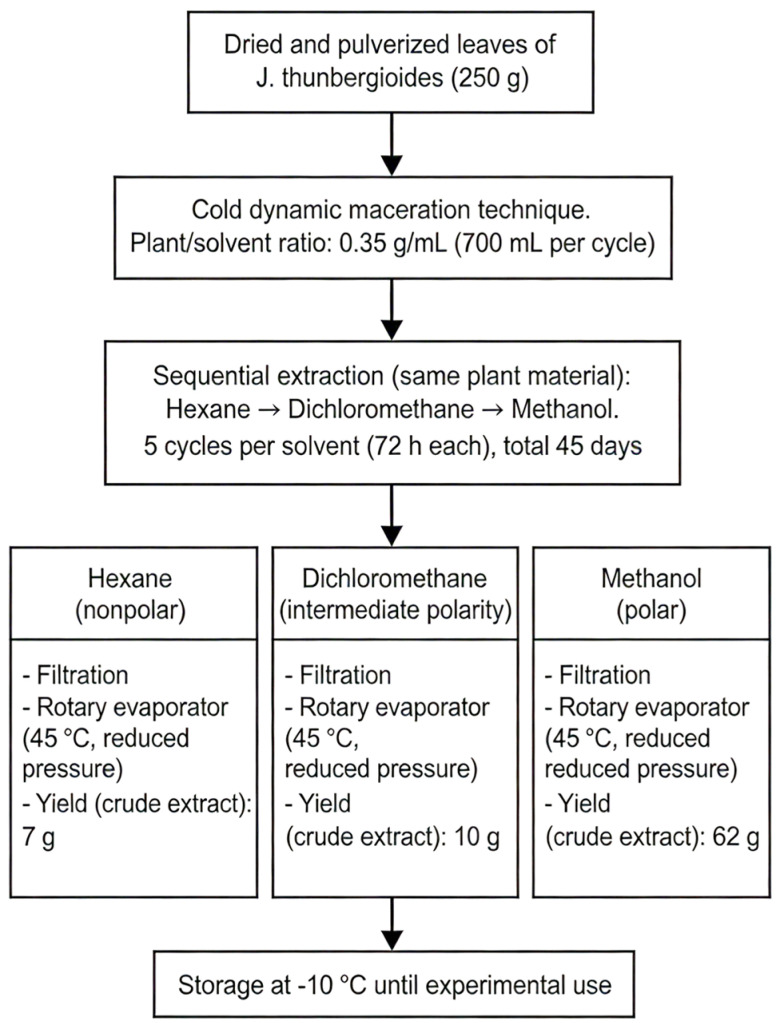
Flowchart illustrating the sequential extraction with increasing solvent polarity of *J. thunbergioides* leaves.

The leaves of the plants were dried in a forced-air circulation oven at 40 °C (modelQ317M42, Quimis, Diadema, SP, Brazil) for seven days and then ground via a rotary knife mill (model TE-650, Tecnal, Piracicaba, SP, Brazil) [36]. A total of 270 g of powdered vegetal material was obtained from *J. thunbergioides*. The resulting powders were properly labeled, packaged, and stored at room temperature until use in the experiments. Extracts were obtained from 250 g of dried and pulverized *J. thunbergioides* leaf material via cold dynamic maceration [37]. A total of five sequential extractions were performed with hexane, dichloromethane, and methanol, with 72-h intervals between each, over a period of 45 days, using the same plant material throughout the process. Each extract was subsequently concentrated under reduced pressure via a rotary evaporator at 45 °C (Figure 11). From the 250 g of dried plant material, the following crude extract yields were obtained: hexane extract (7 g; 2.8% *w*/*w*), dichloromethane extract (10 g; 4.0% *w*/*w*), and methanolic extract (62 g; 24.8% *w*/*w*). The resulting crude extracts were stored in a freezer at −10 °C until use in the experimental procedures.

### 4.3. Preliminary Phytochemical Screening

Phytochemical screening was performed using dried and pulverized *J. thunbergioides* leaves, and specific chemical reaction assays were carried out through adapted protocols [38,39,40]. The analyses aimed to identify the presence of secondary metabolites, including anthraquinone glycosides, terpenes, flavonoids, saponins, tannins, alkaloids, and coumarins. Alkaloids were evaluated through precipitation reactions employing Mayer’s, Dragendorff’s, Bouchardat’s, Bertrand’s, and Hager’s reagents, as well as the tannic acid test. Anthraquinones were screened via the indirect Bornträger test. Flavonoids were identified via Shinoda and oxalo–boric reactions, alongside the sulfuric acid concentration assay. Coumarins were detected by the appearance of fluorescence under UV light after exposure to alkaline solutions. Saponins were screened via the persistent foaming test, which is indicative of their surface-active properties. Tannins were evaluated for their ability to interact with gelatin and metallic salts. Finally, terpenoids were assessed via the Liebermann–Burchard, Pesez, and Keller–Kiliani reactions. The results were denoted as (+) for the presence and (−) for the absence of phytochemicals.

### 4.4. Column Chromatography

Fractionation and purification of the compounds were performed via column liquid chromatography (CC) on silica gel 60 (63–200 µm, Vetec, Rio de Janeiro, RJ, Brazil) as the stationary phase. Columns of varying diameters were employed and eluted with hexane and ethyl acetate under isocratic and/or gradient conditions, and the results were selected according to the physicochemical characteristics of each sample.

### 4.5. Analytical Thin-Layer Chromatography

Fraction analysis was carried out by analytical thin-layer chromatography (TLC) using aluminum-backed Whatman UV 254 plates (0.25 mm thickness) coated with silica gel. Visualization was performed under ultraviolet light at 254 nm, followed by chemical detection via anisaldehyde and potassium permanganate (KMnO_4_) staining reagents [38].

### 4.6. Gas Chromatography–Mass Spectrometry (GC–MS)

Gas chromatography–mass spectrometry (GC–MS) analyses were carried out on a Shimadzu GC-2010 Plus gas chromatograph equipped with a Shimadzu AOC-500 autosampler and coupled to a Shimadzu MS-2010 Plus mass-selective detector operating in electron ionization mode (Shimadzu Corporation, Kyoto, Japan) (EI, 70 eV). Separations were achieved via a DB-5MS capillary column (30 m × 0.25 mm i.d., 0.25 µm film thickness (J&W Scientific, Agilent Technologies, Santa Clara, CA, USA)). The oven temperature was programmed from 60 °C (held for 1 min) to 250 °C at a rate of 5 °C min^−1^. The samples (1 µL) were injected in split mode (1:20). Helium was used as the carrier gas at a constant pressure of 57 kPa. The total run time ranged from 35 to 60 min, depending on the sample analyzed [56]. Blank analyses were conducted under identical GC–MS conditions prior to sample injection. The ethyl acetate used for liquid–liquid partitioning was commercially stabilized with butylated hydroxytoluene (BHT). Ethyl acetate was used solely during fractionation and not in the antioxidant or electrochemical assays.

### 4.7. Fractionation of the Dichloromethane Extract from J. thunbergioides Leaves

Approximately 3 g of the dichloromethane extract obtained from the leaves of *J. thunbergioides* was subjected to selective filtration through a sintered glass funnel packed with silica gel (60 g, 6 nm pore diameter; Acros Organics, Geel, Belgium), following the procedure described [57,58]. Sequential elution afforded the hexane (JTFDH), dichloromethane (JTFDD), ethyl acetate (JTFDA), and methanol (JTFDM) fractions. Each fraction was concentrated under reduced pressure via a rotary evaporator and subsequently evaluated via analytical thin-layer chromatography (TLC). The crude dichloromethane extract and all the fractions were applied with capillary tubes onto aluminum-backed silica gel 60 F_254_ TLC plates and developed via gradient solvent systems of hexane/ethyl acetate (9:1, 7:3, and 1:1, *v*/*v*), dichloromethane/ethyl acetate (9:1 and 7:3, *v*/*v*), and ethyl acetate/methanol (9:1, *v*/*v*). After development, the plates were examined under UV light at 254 and 365 nm and visualized via anisaldehyde and potassium permanganate (KMnO_4_) staining reagents [59,60].

The identification of the compounds was performed by comparison of their mass spectra and retention indices with those reported in the literature and spectral databases [57,61,62]. Relative percentages of the constituents were calculated by peak area normalization, expressed as the percentage of each peak area relative to the total ion chromatogram area. Retention indices (Kovats index, KI) were determined by co-injection of the samples with a homologous series of n-alkanes (C9–C22), analyzed under identical chromatographic conditions. Sample solutions were prepared at a concentration of 1 mg mL^−1^, using dichloromethane for the JTFDD fraction and ethyl acetate for the JTFDA fraction, while the n-alkane standards were prepared at the same concentration in dichloromethane.

### 4.8. Fractionation of the Crude Methanolic Leaf Extract of J. thunbergioides

Acid–base extraction was applied to the methanolic extract (30 g) obtained from the leaves of *J. thunbergioides* using chloroform, with the aim of isolating alkaloids and coumarins. The resulting precipitate was subjected to acid hydrolysis with 1 N HCl at 100 °C for 2 h to promote sugar release. After hydrolysis, the solution was neutralized with NaOH and subsequently partitioned with ethyl acetate, affording fractions F2 and F3, in accordance with previous methods [63]. Fractions F1, F2, and F3 were then analyzed via analytical thin-layer chromatography and gas chromatography–mass spectrometry. The compounds were identified by comparing their mass spectra and retention indices with data reported in the literature.

### 4.9. Antioxidant Activity Assay

The antioxidant activity of the crude extracts of different polarities (hexane, dichloromethane, and methanol) was assessed through the scavenging of the free radical 2,2-Diphenyl-1-picrylhydrazyl (DPPH). The procedure involved the preparation of a DPPH stock solution in ethanol (0.1 mmol), from which 1 mL was added to 1 mL of the solutions containing the crude extracts at varying concentrations [41].

Methanolic extract solutions of *J. thunbergioides* at concentrations of 1, 2, 4, 6, 8, 10, 16, 22, and 32 μg·mL^−1^ were prepared from a stock solution of 1600 μg·mL^−1^. The final mixtures contained 0.05 mmol of DPPH and 0.5, 1, 2, 3, 4, 5, 8, 12, or 16 μg·mL^−1^ methanolic extract from the leaves of *J. thunbergioides*. For the dichloromethane extract, final concentrations of 10, 20, 30, 40, 60, 125, 190, and 250 μg·mL^−1^ were prepared from a stock solution of 2140 μg·mL^−1^. For the hexane extract, final concentrations of 10, 25, 50, 125, 250, 400, and 500 μg·mL^−1^ were prepared from a stock solution of 2500 μg·mL^−1^. The mixtures were stirred at 450 rpm for 30 min and kept in a dark environment at room temperature (25 °C). DPPH• absorbs visible light maximally at 517 nm, and its characteristic purple color fades upon reduction by an antioxidant.

The absorbance readings were performed via a UV–VIS spectrophotometer (Shimadzu Corporation, Kyoto, Japan). A solution containing 1 mL of DPPH and 1 mL of ethanol was used as the blank (negative control), while a solution containing 10 µmol of gallic acid served as the positive control [42,43]. The percentage of radical scavenging activity was calculated via Equation (1).

DPPH scavenging percentage (%) = [Abs blank − Abs sample] × 100 (1)

Abs blank

The IC_50_ (concentration causing 50% inhibition) was determined graphically via a linear calibration curve by plotting the extract concentrations vs. the associated scavenging action. The antioxidant activity index (AAI) was calculated via Equation (2) [44], considering that 0.5 mmol corresponds to 19.71 µg·mL^−1^ of DPPH:

AAI = DPPH final concentration (μg·mL^−1^) (2)

IC_50_ (μg·mL^−1^)

### 4.10. Electrochemical Analysis

Voltammetric experiments were carried out in a potentiostat/galvanostat (PGSTAT, model 204, Metrohm Autolab, Utrecht, The Netherlands) integrated with NOVA 2.1 software (Metrohm Autolab, Utrecht, The Netherlands). The measurements were made in a 5 mL single-compartment electrochemical cell with a 3-electrode system consisting of a carbon paste electrode, an Ag/AgCl/KCl salt 3 M electrode, and a platinum wire (purchased from Lab Solutions, SP, Brazil), representing the working, reference, and auxiliary electrodes, respectively [45]. The experimental conditions for differential pulse voltammetry (DPV) were a pulse amplitude of 50 mV, pulse width of 0.5 s and scan rate of 10 mV·s^−1^. The experimental conditions for the square wave voltammetry (SWV) were a pulse amplitude of 50 mV with a frequency of 50 Hz and a potential increase of 2 mV, corresponding to a scanning rate of 100 mV·s^−1^. The experimental conditions for cyclic voltammetry were a scan range from 0.2 to 1 V and a scan rate of 100 mV·s^−1^. The employed scan rate was selected to minimize the adsorption of oxidized species on the electrode surface, thus providing reproducible results. All the voltammograms were corrected via the baseline-corrected method, and all the data were analyzed via Origin 8.0 software (OriginLab Corporation, Northampton, MA, USA) [46,47]. All the experiments were performed with 10 µg·mL^−1^ methanolic extract at room temperature (21 ± 1 °C), and the main electrolyte used was 0.1 M phosphate buffer, pH 7.0.

## 5. Conclusions

The present study demonstrates that *Justicia thunbergioides* leaves constitute a relevant source of bioactive secondary metabolites, including alkaloids, flavonoids, terpenoids, and tannins, which collectively contribute to their pronounced antioxidant potential. Among the evaluated samples, the methanolic extract exhibited the most consistent and concentration-dependent free radical scavenging activity, reaching levels comparable to gallic acid. Electrochemical analyses further revealed that the methanolic extract displays a well-defined redox profile, characterized by partially reversible electron transfer processes, which is indicative of the presence of redox-active phenolic constituents. These electrochemical features, together with the diversity of antioxidant metabolites identified by GC–MS, provide complementary and convergent evidence supporting the strong antioxidant profile of *J. thunbergioides*. Overall, the results reinforce the relevance of *J. thunbergioides* as a promising natural source of redox-active metabolites and support its potential applicability in studies targeting oxidative stress–related processes.

## Figures and Tables

**Table 1:** Phytochemical screening of *Justicia thunbergioides* leaves.

Phytochemical Compounds	*J. thunbergioides*
Alkaloids	++++++ ^1^
Anthraquinones	−
Coumarins	+
Flavonoids	+++
Saponins	+
Tannins	++
Terpenoids	+++

^1^ (+) indicates presence, and (−) indicates absence. Multiple (+) symbols denote the quantity of tests indicating the existence of a phytochemical group.

**Table 2:** Chemical composition of the ethyl acetate fraction obtained from the dichloromethane extract of *J. thunbergioides*.

Peak	RT/min	RI	Compounds	Relative Content (%)
1	9.12	1000	Decane	0.31
2	10.32	1032	1,8-cineole	0.30
3	14.06	1133	Cyclooctanone	1.27
4	16.15	1189	*p*-cymen-8-ol	0.57
5	19.25	1276	2,6-dimethyl-octa-1,7-dien-3,6-diol	4.42
9	21.98	1356	trans-*p*-menth-6-en-2,8-diol	4.41
12	23.85	1413	NI ^1^	33.80
15	24.54	1435	8-Hydroxycarvotanacetone	0.69
24	28.94	1578	Spathulenol	0.77
26	33.39	1736	Oplopanone	2.87
35	56.27	2905	1-hexacosanol	0.58
37	58.27	3108	octacosanol	2.01
38	58.50	3129	α-tocopherol	0.45
39	59.00	3171	Sesamin	1.27
40	60.99	3320	*β*-sitosterol	2.09

NI ^1^: not identified. RT: retention time. RI: experimental retention index.

**Table 3:** Chemical composition of the dichloromethane fraction obtained from the dichloromethane extract of *J. thunbergioides*.

Peak	RT/min	RI	Compounds	Relative Content (%)
1	18.04	1242	Ascaridole	19.81
2	20.33	1307	Isoascaridole	26.38
3	29.73	1605	β-Oplopenone	17.26
4	44.91	2210	Octadecanol acetate	1.02
5	50.92	2510	Pentacosane	0.63
6	51.45	2531	Bis(2-ethylhexyl) phthalate	5.49
7	52.69	2600	Hexacosane	1.11
8	54.08	2700	Heptacosane	1.24
9	55.34	2811	Squalene	0.75
10	56.22	2900	Nonacosane	3.53
11	57.59	3043	Octacosanal	9.04
12	58.68	3144	Nonacosanal	0.67
13	59.16	3185	NI ^1^	2.47
14	59.95	3246	Triacontanal	10.60

NI ^1^: not identified. RT: retention time. RI: experimental retention index.

**Table 4:** Antioxidant activity of different leaf extracts from *J. thunbergioides*.

Extract	DPPH (IC_50 μg·mL_^−1^)	AAI
Methanolic	3.2 ^1^	6.15
Dichloromethanic	78.5	0.25
Hexanic	186.3	0.10
Galic acid	4.2	4.69

^1^ IC_50_: concentration required to inhibit 50% (IC_50_) of the DPPH radical. AAI: antioxidant activity index. Galic acid (10 µmol) was used as a positive control.

## Data Availability

The original contributions presented in this study are included in the article/Supplementary Material. Further inquiries can be directed to the corresponding author.

## Supplementary Materials

The following supporting information can be downloaded at https://www.mdpi.com/article/10.3390/ph19030486/s1: Figure S1. GC-MS. Mass spectrum of (a1) decane (peak 1); (b1) 1,8-cineole (peak 2); (c1) cyclooctanone (peak 3); (d1) *p*-cymen-8-ol (peak 4); (e1) 2,6-dimethyl-octa-1,7-dien-3,6-diol (peak 5); (f1) trans-*p*-menth-6-en-2,8-diol (peak 9); (g1) 8-Hydroxycarvotanacetone (peak 15); (h1) spathulenol (peak 24); (i1) oplopanone (peak 26); (j1) 1-hexacosanol (peak 35); (k1) 1-octacosanol (peak 37); (l1) α-tocopherol peak (38); (m1) Sesamin peak (39); (n1) *β*-sitosterol peak (40) of the ethyl acetate fraction obtained from the dichloromethane extract of *J. thunbergioides*. Figure S2. GC-MS. Mass spectrum of (a1)ascaridole (peak 1); (b1) isoascaridole (peak 2); (c1) β-Oplopenone (peak 3); (d1) octadecanol acetate (peak 4); (e1) pentacosane (peak 5); (f1) Bis(2-ethylhexyl) phthalate (peak 6); (g1) hexacosane (peak 7); (h1) heptacosane (peak 8); (i1) squalene (peak 9); (j1) nonacosane (peak 10); (k1) octacosanal (peak 11); (l1) nonacosanal (peak 12); (m1) triacontanal (peak 14) of the dichloromethane fraction obtained from the dichloromethane extract of *J. thunbergioides*.

## Abbreviations

The following abbreviations are used in this manuscript:

DPPH	2,2-diphenyl-1-picrylhydrazyl
WHO	World Health Organization
ROS	Reactive oxygen species
AAI	Antioxidant activity index
GC–MS	Gas chromatography–mass spectrometry
