# Supplementary material for: Phytochemical Profiling and Antioxidant Activity of Justicia thunbergioides (Lindau) Leonard (Acanthaceae): A Promising Source of Therapeutic Metabolites
Source: Pharmaceuticals (Basel). 2026 Mar 16;19(3):486. doi: 10.3390/ph19030486 (PMC13028939; doi:10.3390/ph19030486)
Supplement: Supplementary file 1 [file pharmaceuticals-19-00486-s001.zip › pharmaceuticals-4185792-supplementary.pdf]

SUPPLEMENTARY INFORMATION

Article - ***Phytochemical Profiling and Antioxidant Activity of Justicia thunbergioides (Lindau) Leonard (Acanthaceae): A Promising Source of Therapeutic Metabolites***

a1) Experimental mass spectrum of decane (peak 1)

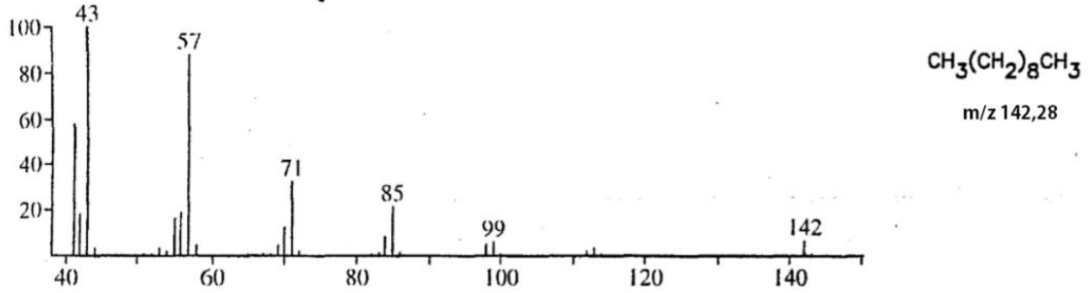

a2) Library mass spectrum of decane

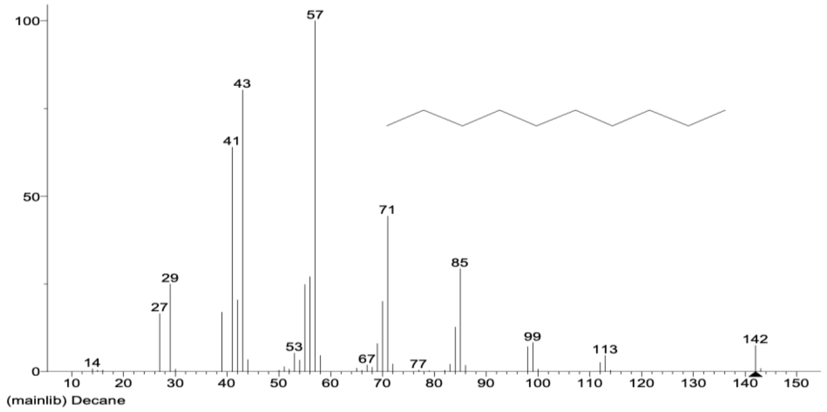

b1) Experimental mass spectrum of 1,8-cineole (peak 2)

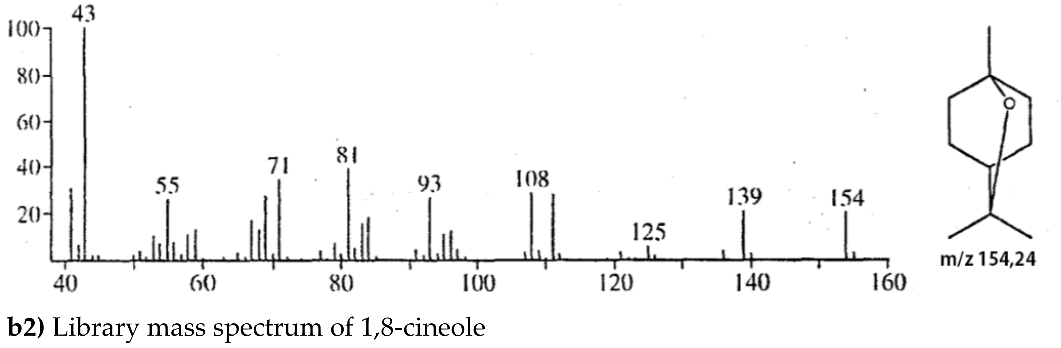

b2) Library mass spectrum of 1,8-cineole

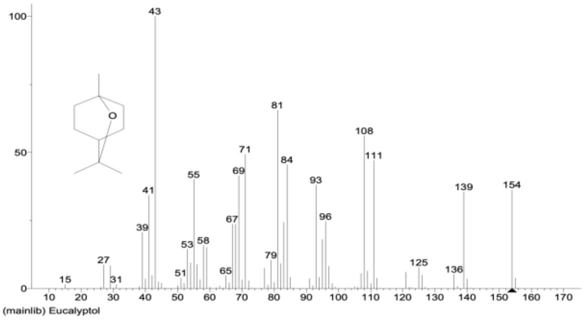

c1) Experimental mass spectrum of cyclooctanone (peak 3)

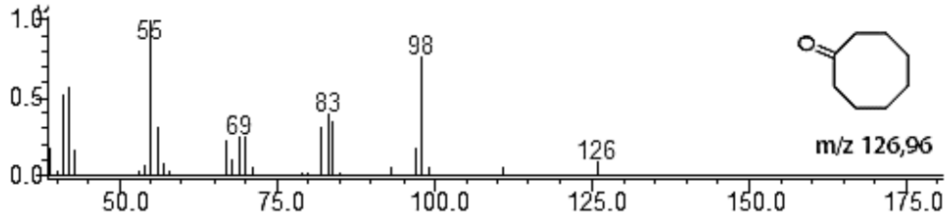

c2) Library mass spectrum of cyclooctanone

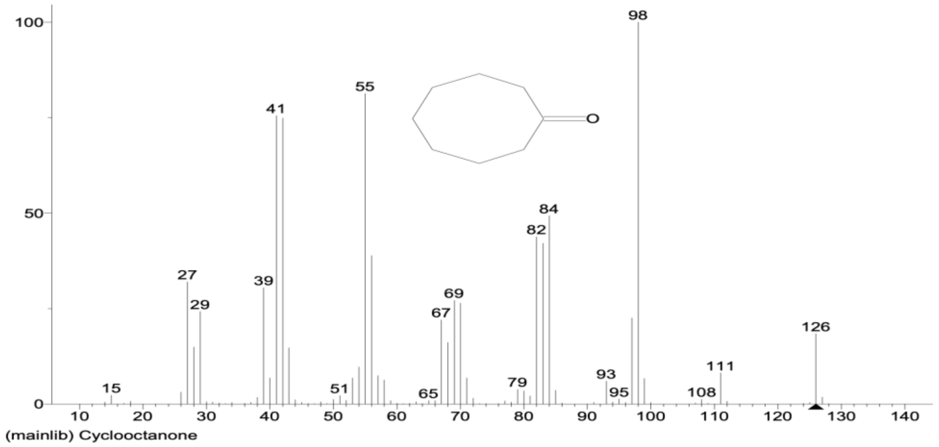

d1) Experimental mass spectrum of *p*-cymen-8-ol (peak 4)

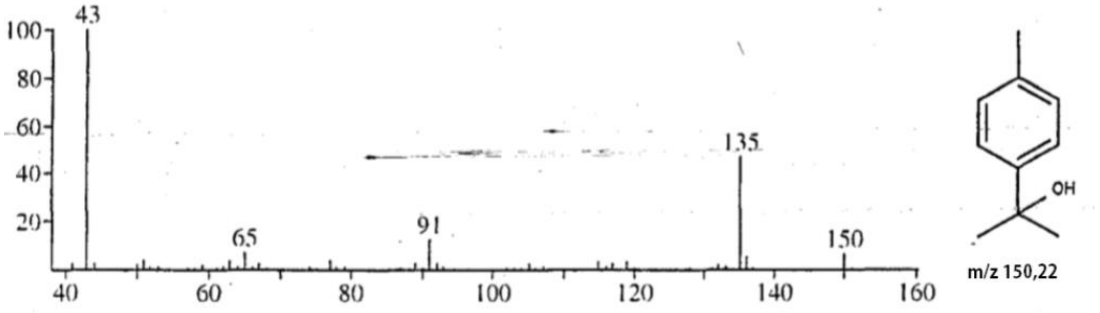

d2) Library mass spectrum of *p*-cymen-8-ol

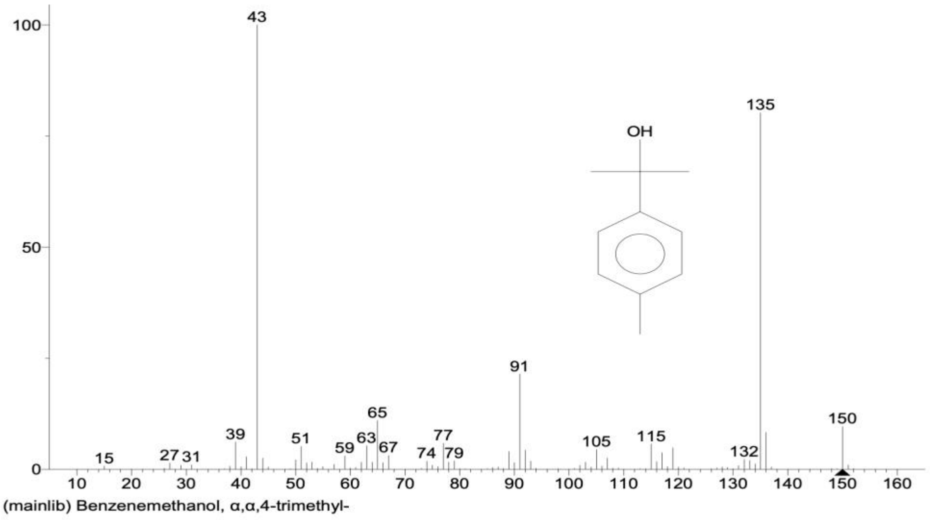

e1) Experimental mass spectrum of 2,6-dimethyl-octa-1,7-dien-3,6-diol (peak 5)

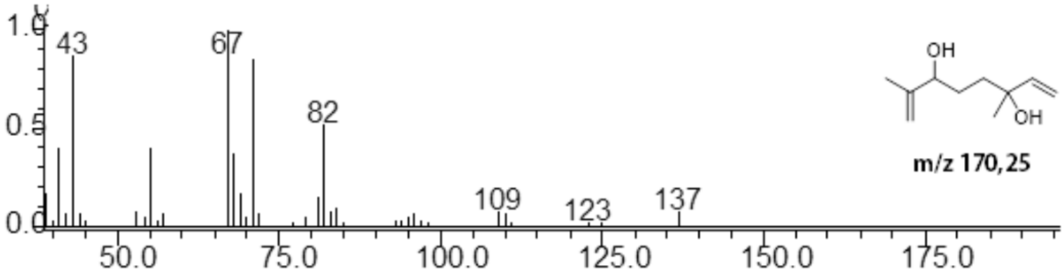

e2) Library mass spectrum of 2,6-dimethyl-octa-1,7-dien-3,6-diol

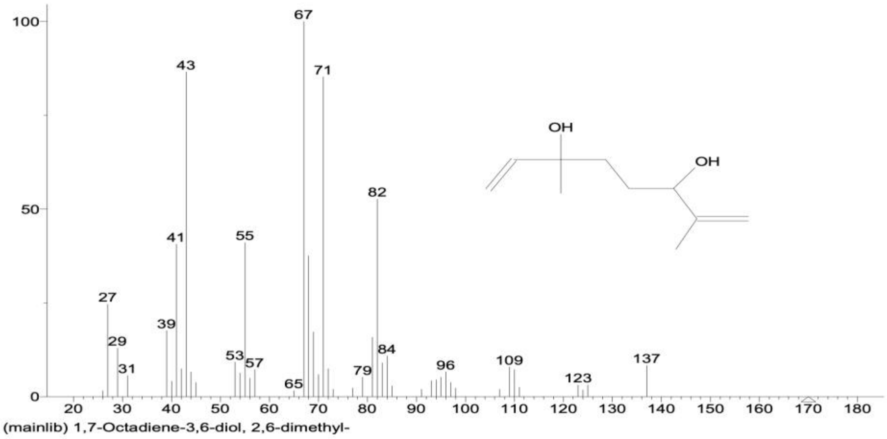

f1) Experimental mass spectrum of *trans-p*-menth-6-en-2,8-diol (peak 9)

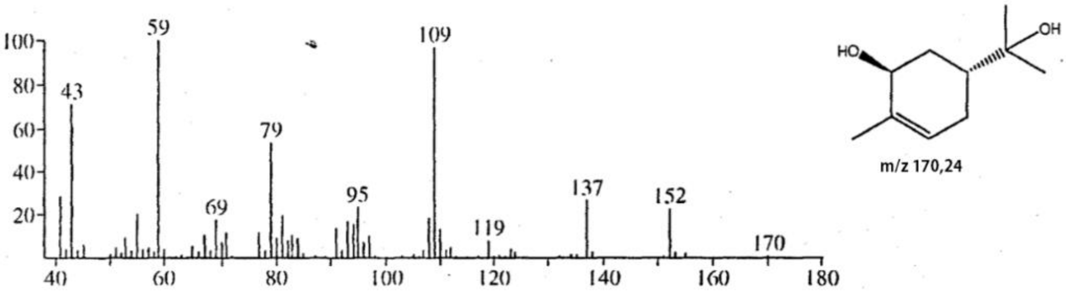

f2) Library mass spectrum of *trans-p*-menth-6-en-2,8-diol

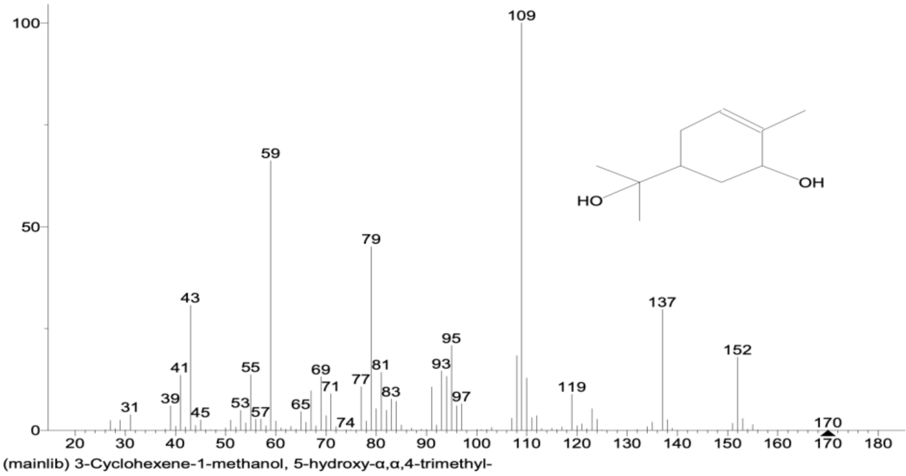

g1) Experimental mass spectrum of 8-hydroxycarvotanacetone (peak 15)

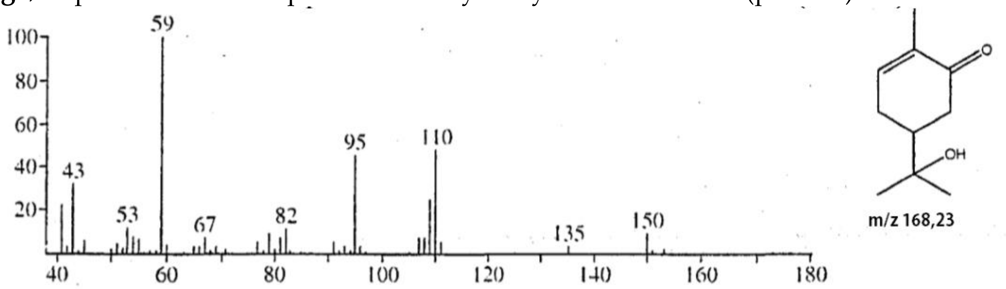

g2) Library mass spectrum of 8-hydroxycarvotanacetone

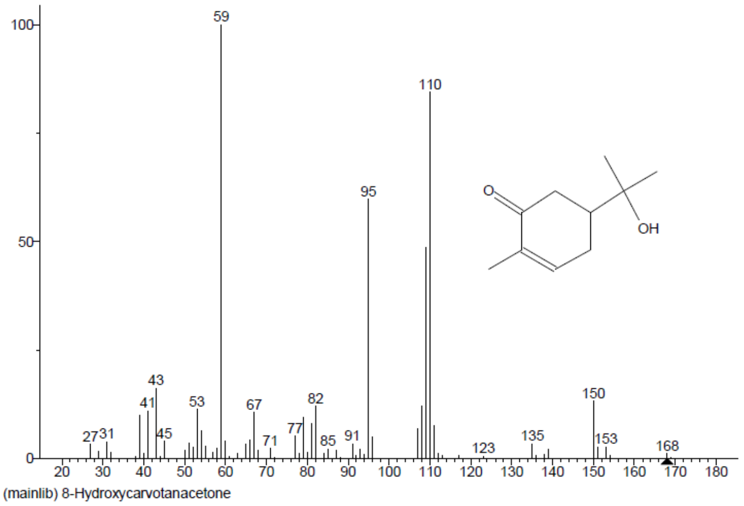

h1) Experimental mass spectrum of spathulenol (peak 24)

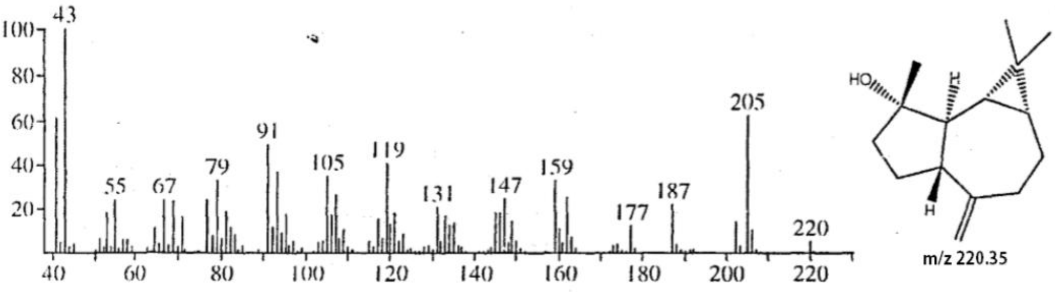

h2) Library mass spectrum of spathulenol

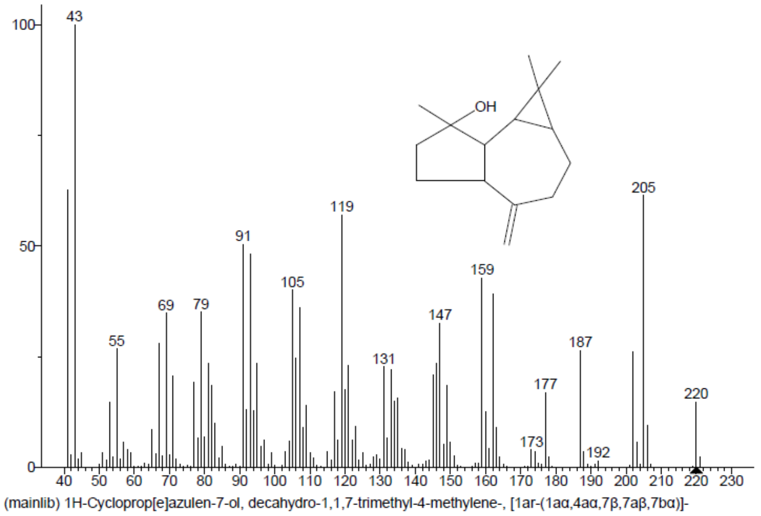

i1) Experimental mass spectrum of oplopanone (peak 26)

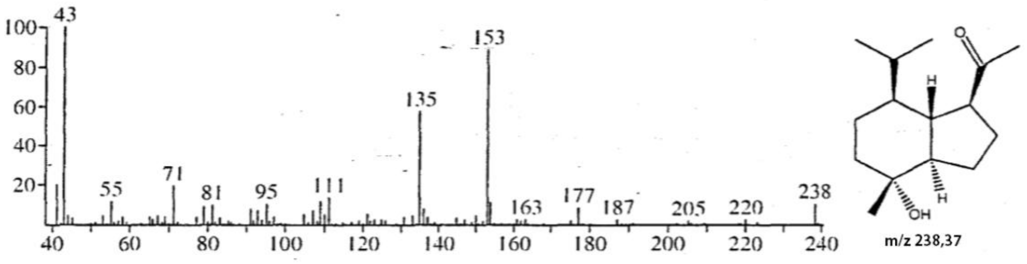

i2) Library mass spectrum of oplopanone

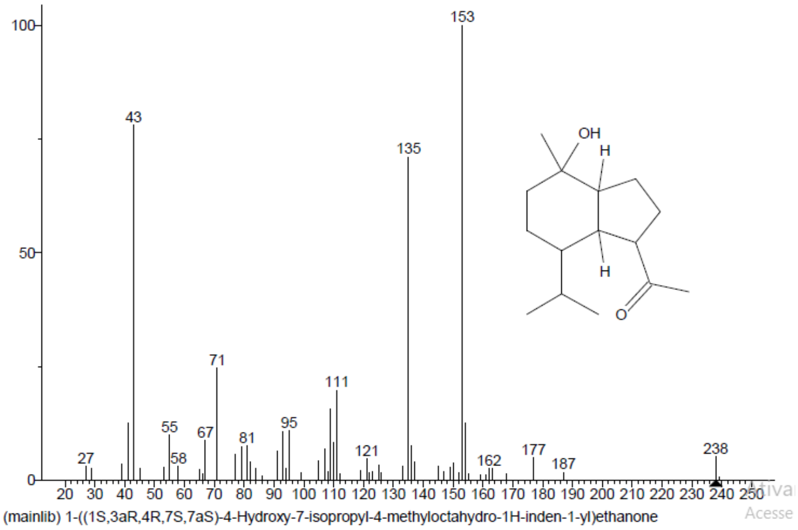

j1) Experimental mass spectrum of 1-hexacosanol (peak 35)

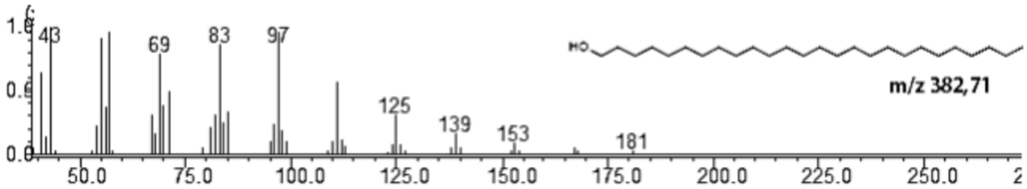

j2) Library mass spectrum of 1-hexacosanol

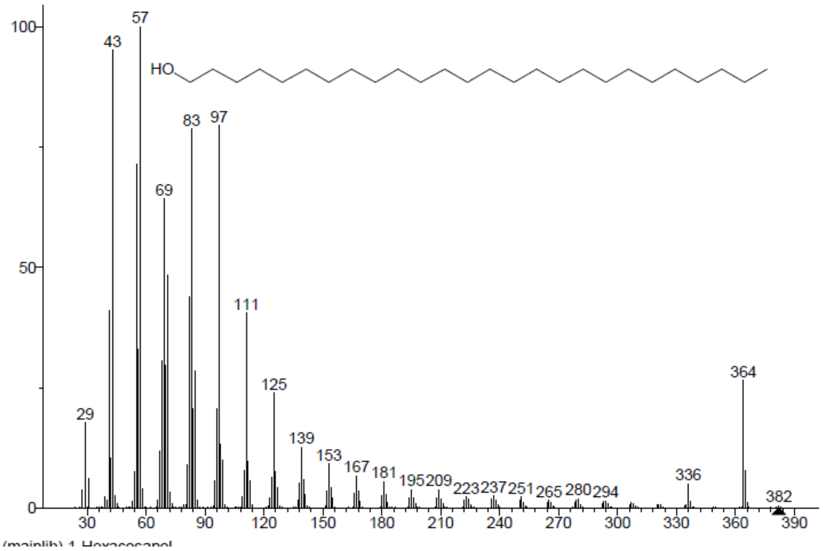

k1) Experimental mass spectrum of 1-octacosanol (peak 37)

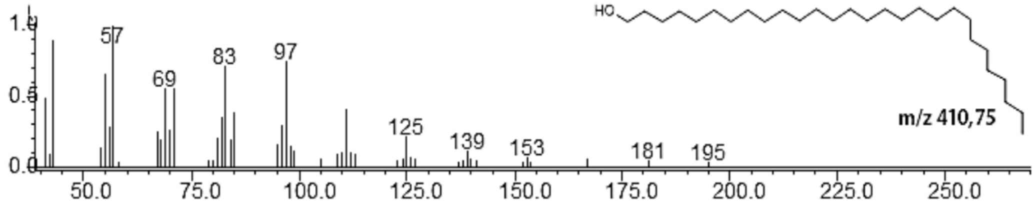

k2) Library mass spectrum of 1-octacosanol

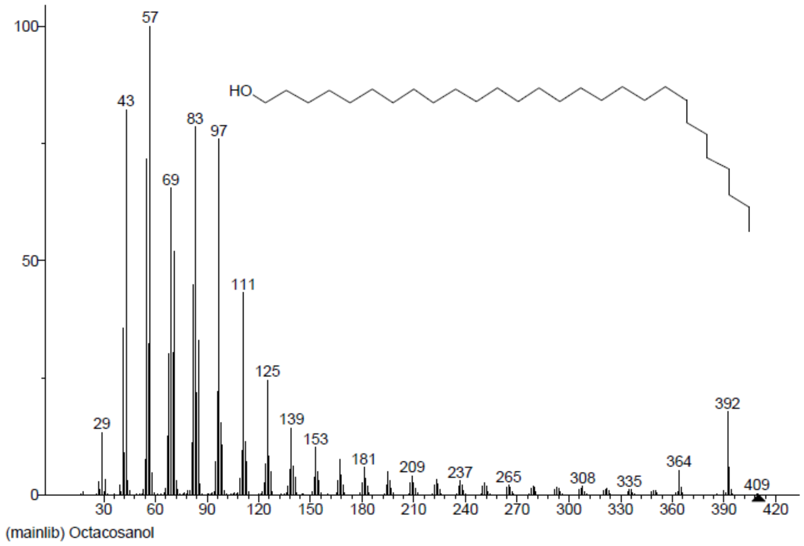

l1) Experimental mass spectrum of α-tocopherol (peak 38)

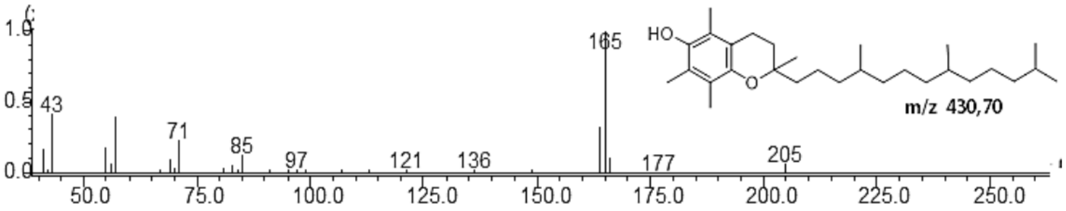

l2) Library mass spectrum of α-tocopherol

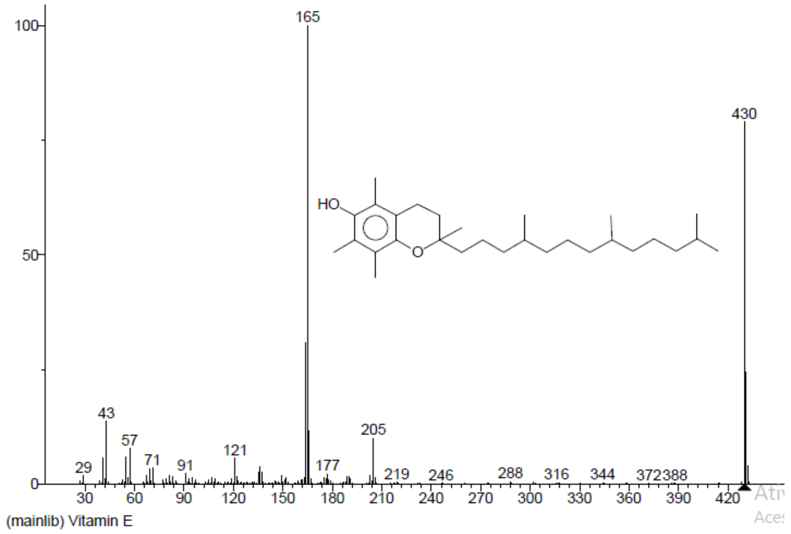

m1) Experimental mass spectrum of sesamin (peak 39)

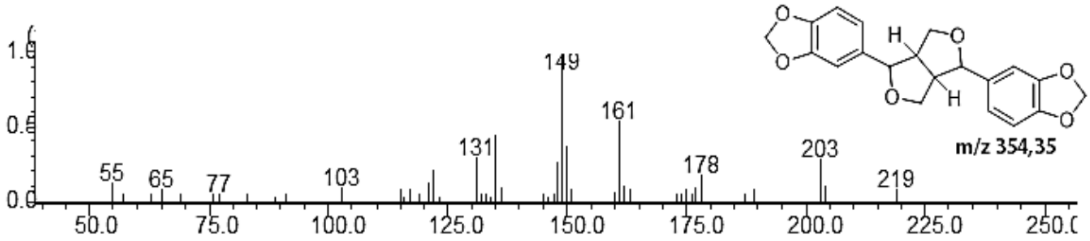

m2) Library mass spectrum of sesamin

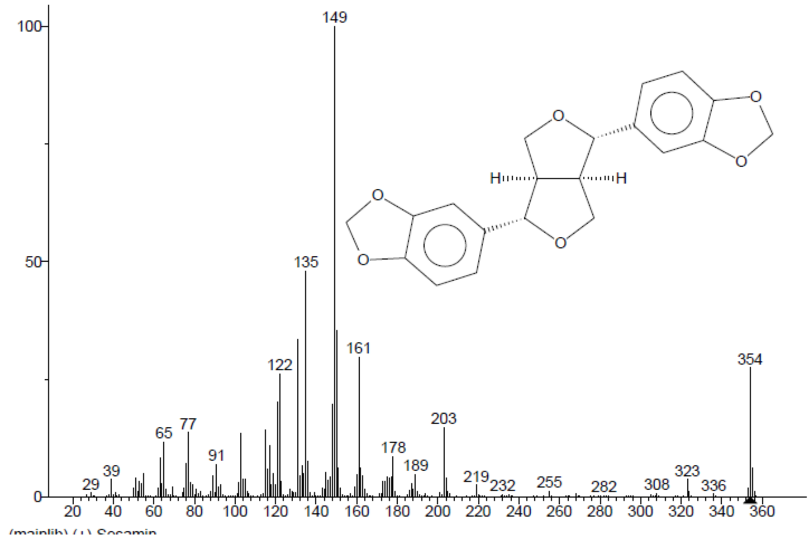

n1) Experimental mass spectrum of *β*-sitosterol (peak 40)

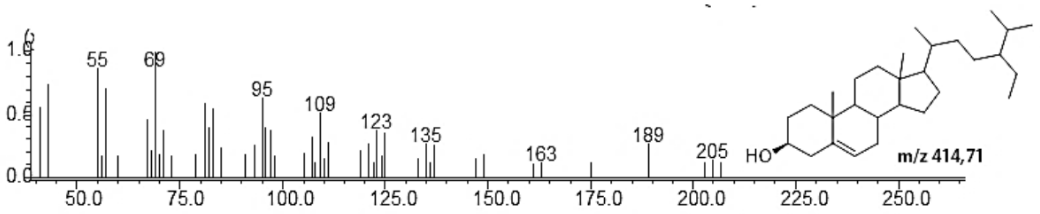

n2) Library mass spectrum of *β*-sitosterol

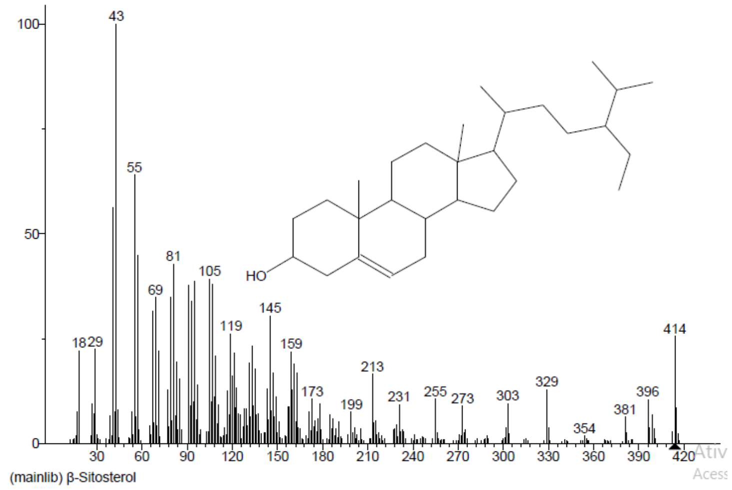

Figure S1. GC–MS mass spectra of compounds identified in the ethyl acetate fraction obtained from the dichloromethane extract of *Justicia thunbergioides*. For each compound, the experimental mass spectrum (**x1**) obtained from the chromatographic peak is shown together with the corresponding library mass spectrum (**x2**) from the GC–MS database used for spectral comparison and compound identification. Compounds: (**a**) decane (peak 1); (**b**) 1,8-cineole (peak 2); (**c**) cyclooctanone (peak 3); (**d**) *p*-cymen-8-ol (peak 4); (**e**) 2,6-dimethyl-octa-1,7-dien-3,6-diol (peak 5); (**f**) *trans-p*-menth-6-en-2,8-diol (peak 9); (**g**) 8-hydroxycarvotanacetone (peak 15); (**h**) spathulenol (peak 24); (**i**) oplopanone (peak 26); (**j**) 1-hexacosanol (peak 35); (**k**) 1-octacosanol (peak 37); (**l**) α-tocopherol (peak 38); (**m**) sesamin (peak 39); and (**n**) β-sitosterol (peak 40).

a1) Experimental mass spectrum of ascaridole (peak 1)

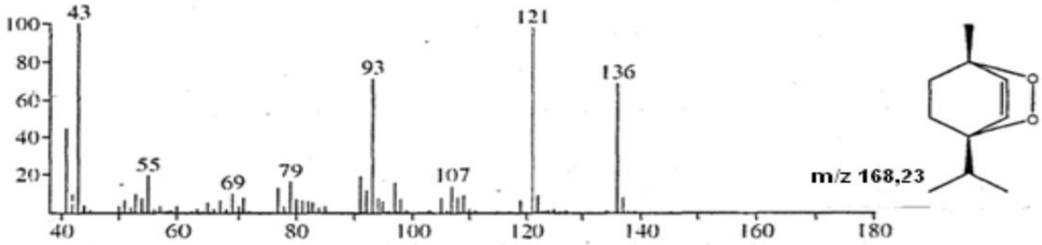

a2) Library mass spectrum of ascaridole

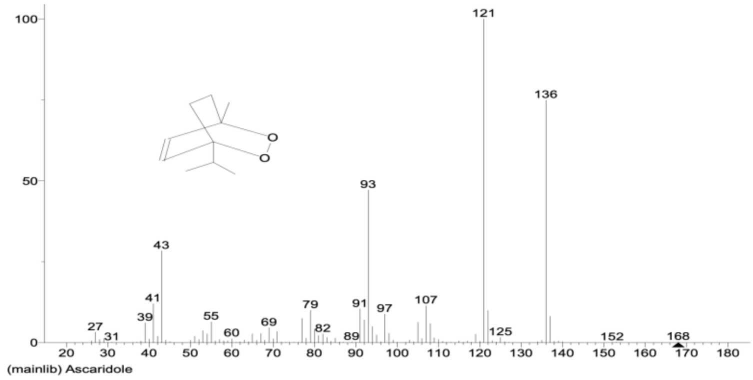

b1) Experimental mass spectrum of isoascaridole (peak 2)

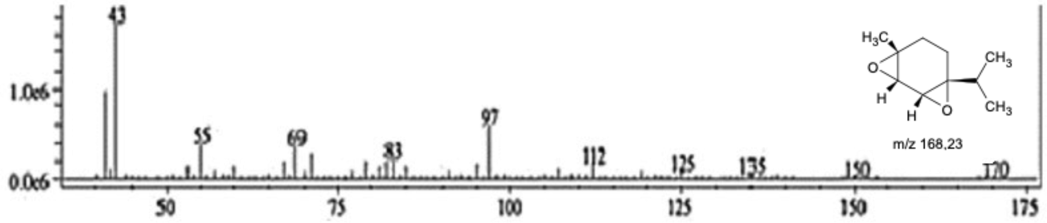

b2) Library mass spectrum of isoascaridole

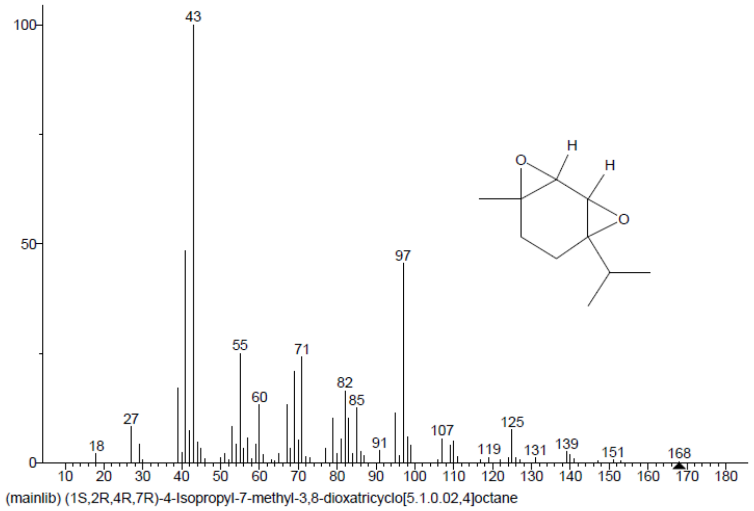

c1) Experimental mass spectrum of β-oplopanone (peak 3)

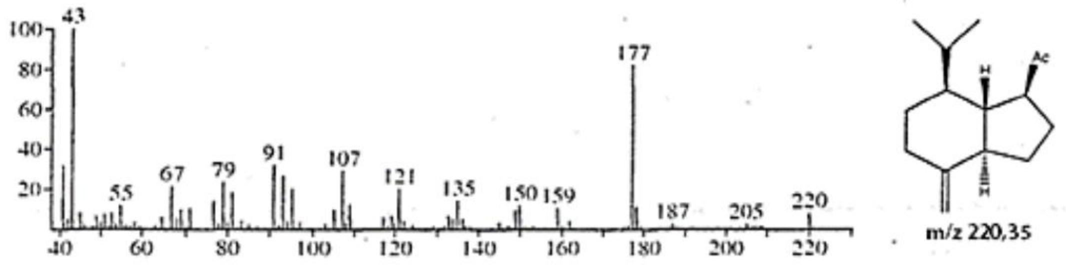

c2) Library mass spectrum of β-oplopanone

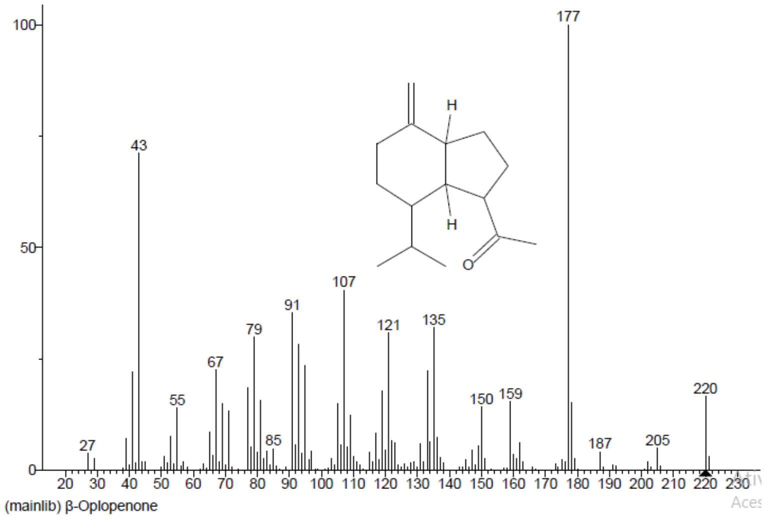

d1) Experimental mass spectrum of octadecanol acetate (peak 4);

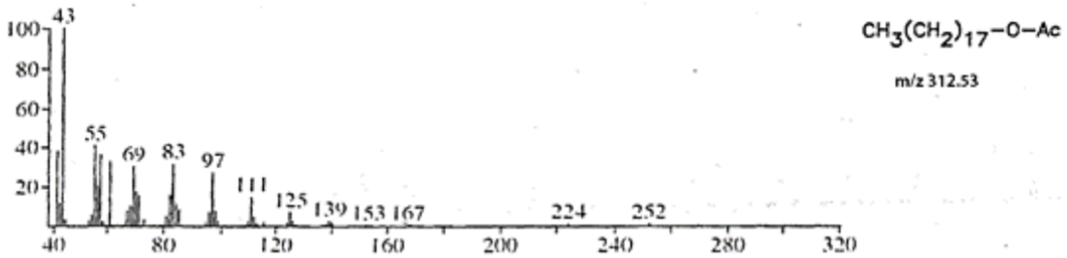

d2) Library mass spectrum of octadecanol acetate

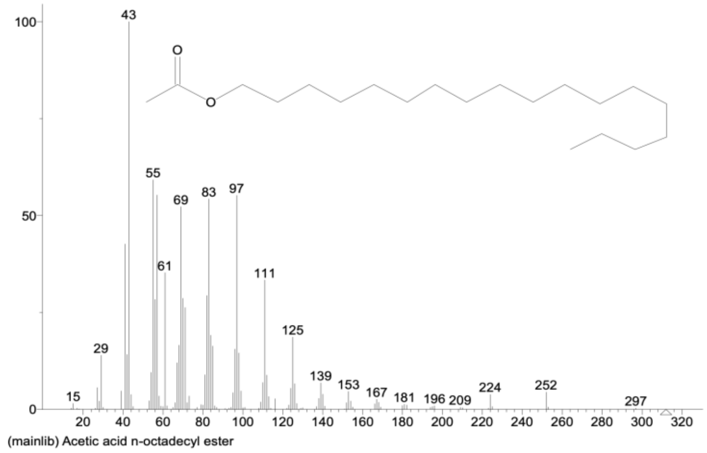

e1) Experimental mass spectrum of pentacosane (peak 5)

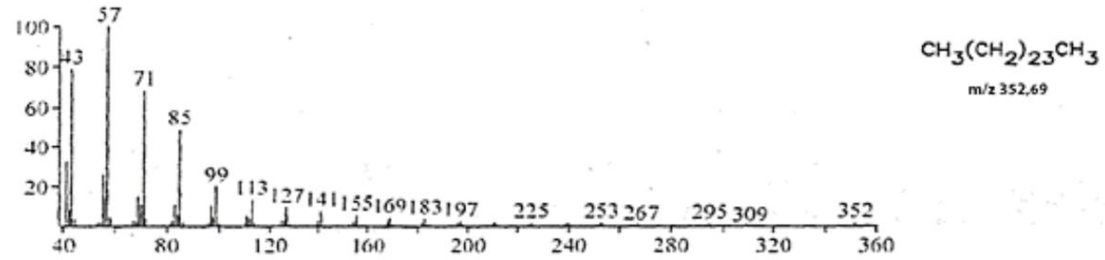

e2) Library mass spectrum of pentacosane

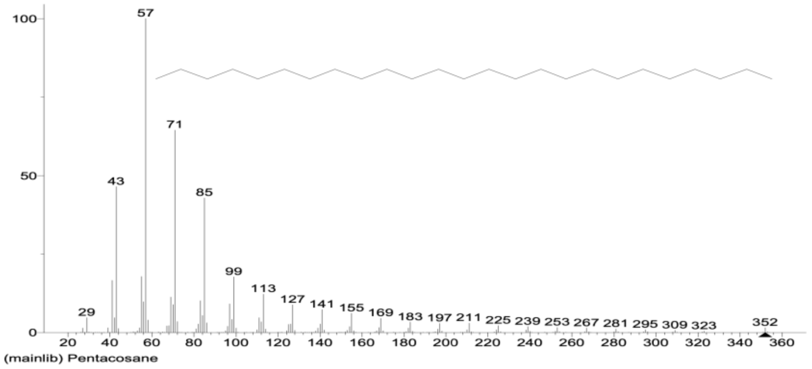

f1) Experimental mass spectrum of *bis*(2-ethylhexyl) phthalate (peak 6)

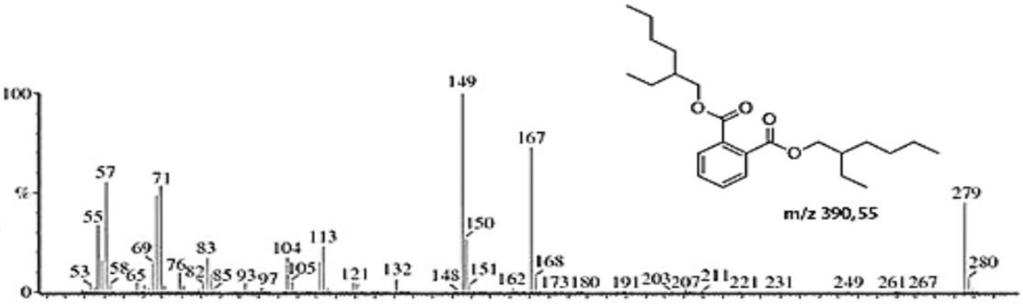

f2) Library mass spectrum of *bis*(2-ethylhexyl) phthalate

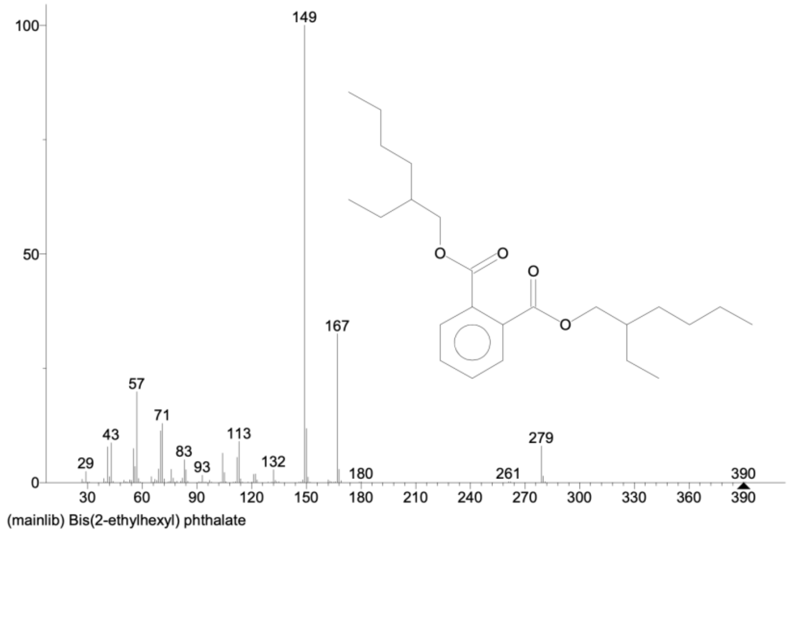

g1) Experimental mass spectrum of hexacosane (peak 7)

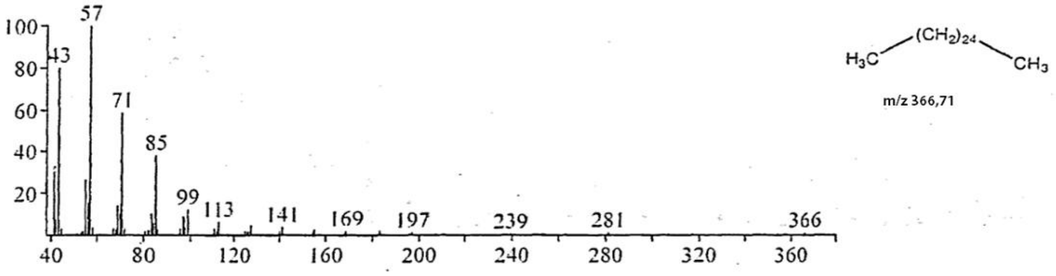

g2) Library mass spectrum of hexacosane

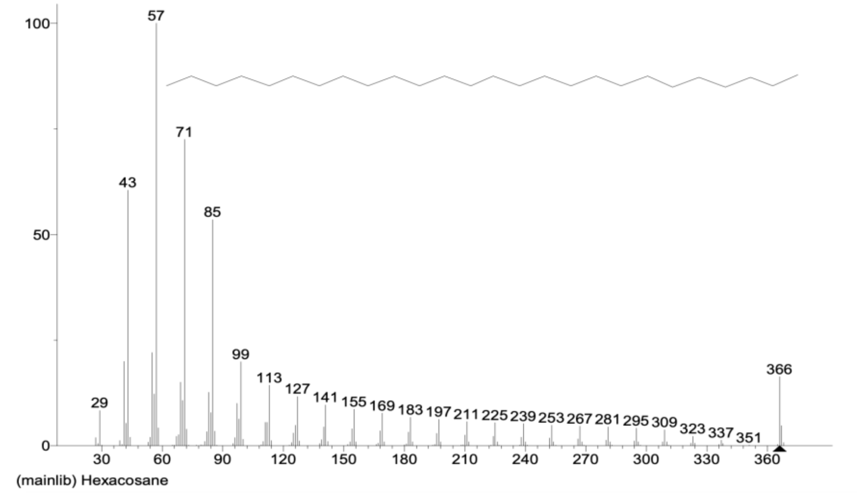

h1) Experimental mass spectrum of heptacosane (peak 8);

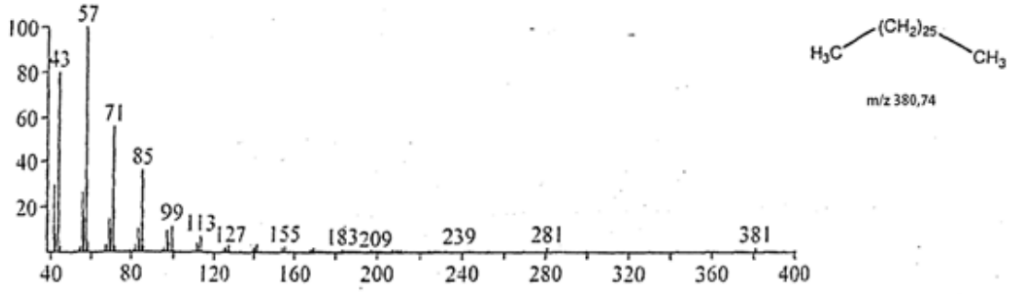

h2) Library mass spectrum of heptacosane

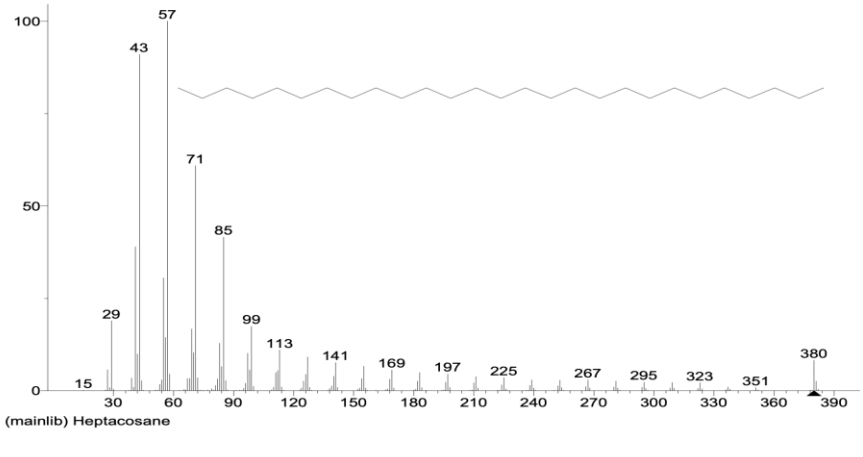

i1) Experimental mass spectrum of squalene (peak 9)

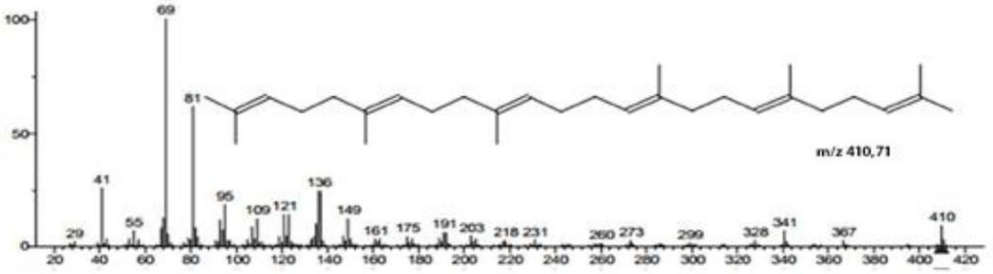

i2) Library mass spectrum of squalene

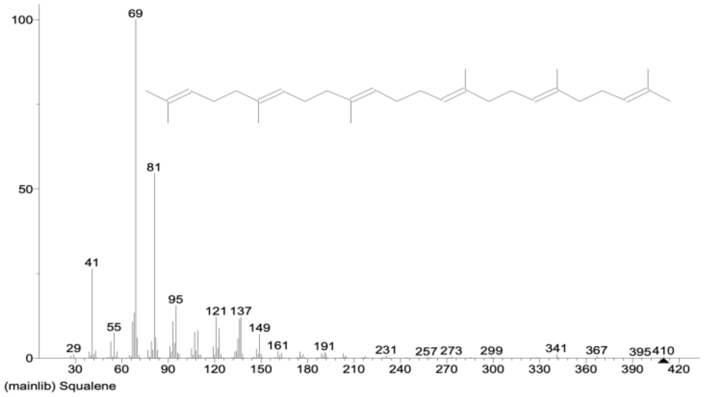

j1) Experimental mass spectrum of nonacosane (peak 10**)**

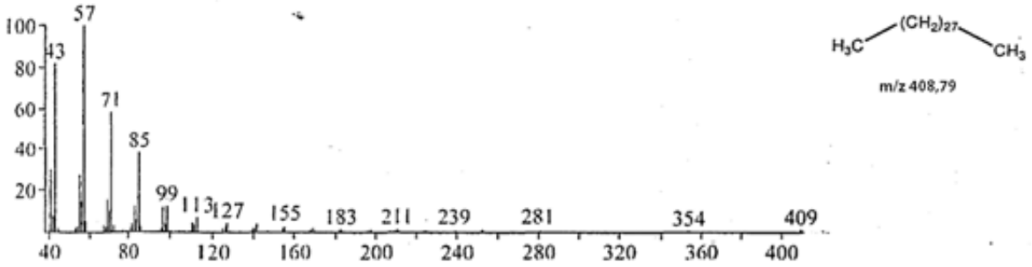

j2) Library mass spectrum of nonacosane

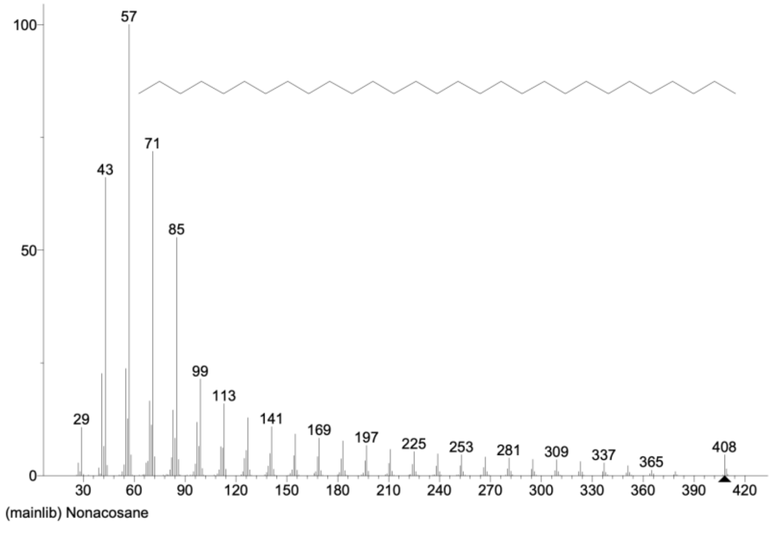

k1) Experimental mass spectrum of octacosanal (peak 11);

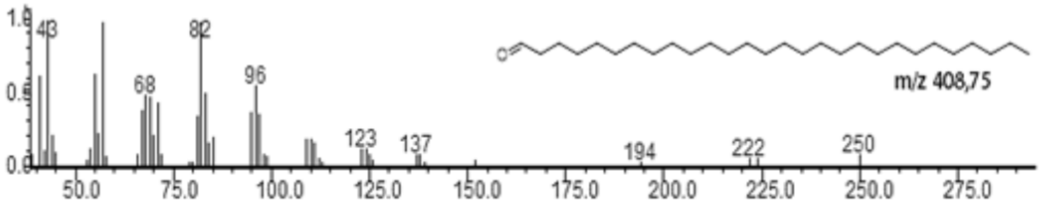

k2) Library mass spectrum of octacosanal

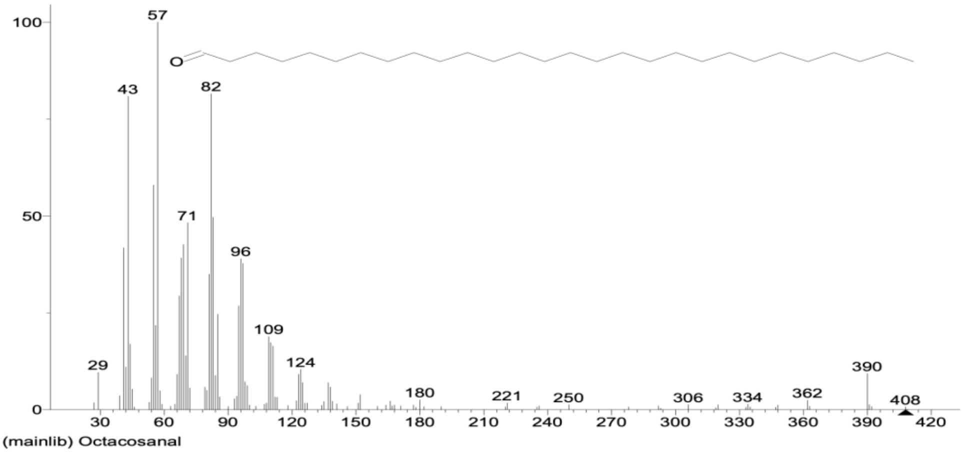

l1) Experimental mass spectrum of nonacosanal (peak 12)

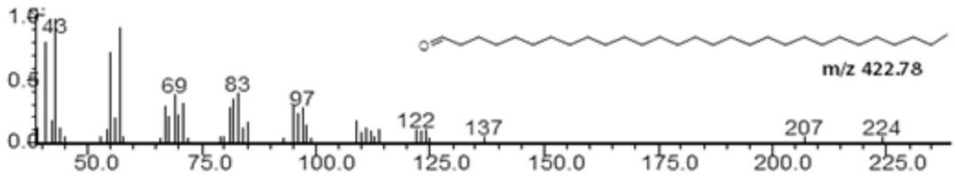

l2) Library mass spectrum of nonacosanal

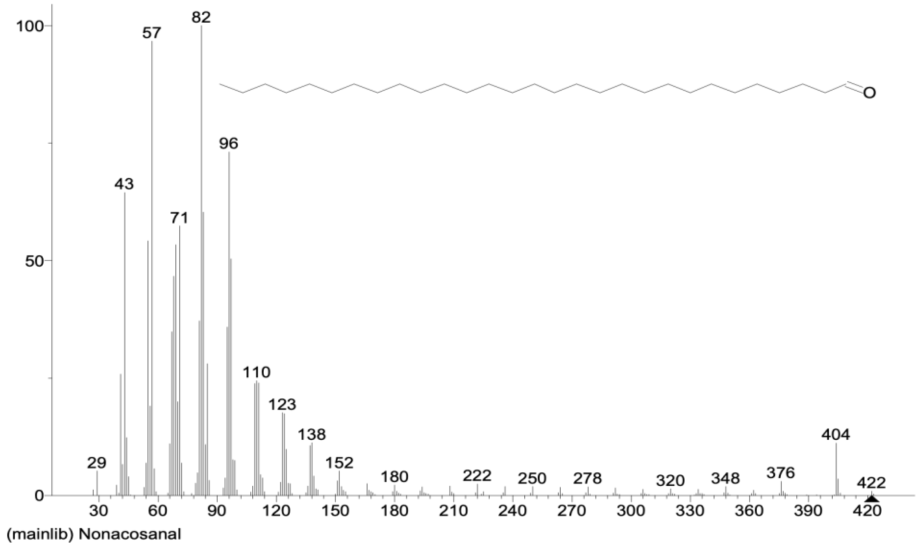

m1) Experimental mass spectrum of triacontanal (peak 14).

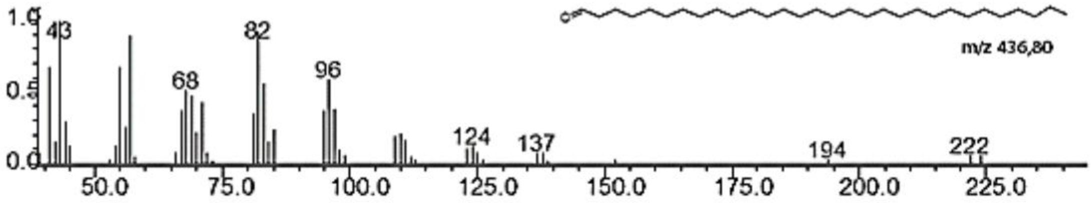

m2) Library mass spectrum of triacontanal

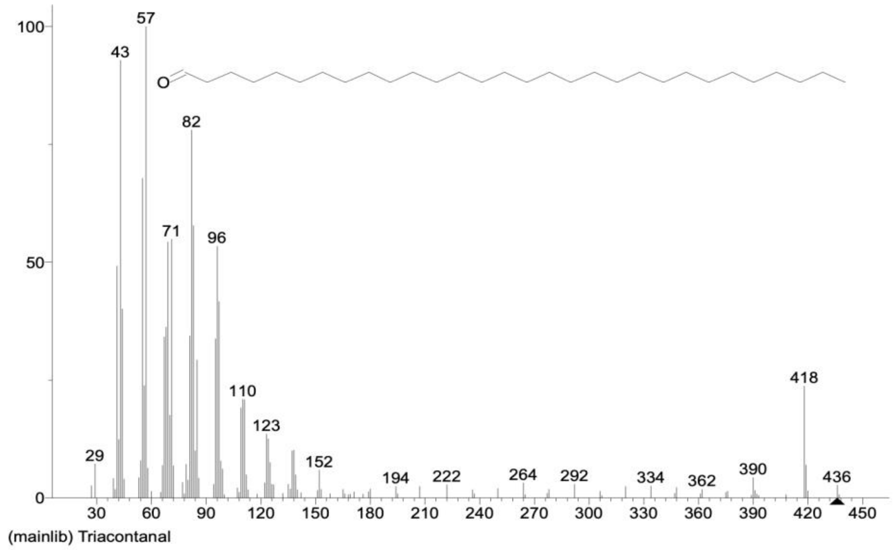

Figure S2. GC–MS mass spectra of compounds detected in the dichloromethane fraction obtained from the dichloromethane extract of *Justicia thunbergioides*. For each metabolite, the experimental spectrum (**x1**) is presented alongside the corresponding library spectrum (**x2**) used for spectral matching and compound identification. Compounds: (**a**) ascaridole (peak 1); (**b**) isoascaridole (peak 2); (**c**) β-oplopanone (peak 3); (**d**) octadecanol acetate (peak 4); (**e**) pentacosane (peak 5); (**f**) bis(2-ethylhexyl) phthalate (peak 6); (**g**) hexacosane (peak 7); (**h**) heptacosane (peak 8); (**i**) squalene (peak 9); (**j**) nonacosane (peak 10); (**k**) octacosanal (peak 11); (**l**) nonacosanal (peak 12); and (**m**) triacontanal (peak 14).
